# Integrating machine learning and geospatial technologies for spatio-temporal analysis of urban expansion and land use transformation in Gambella Town, Ethiopia

**DOI:** 10.1371/journal.pone.0360419

**Published:** 2026-10-20

**Authors:** Kanenus Fufa Dararo, Goanar Jal Dup, Weyesa Merga Chemeda, Shankar Karuppannan

**Affiliations:** 1 Department of Spatial Decision Support, Space Science and Geospatial Institute, Addis Ababa, Ethiopia; 2 Department of Geography and Environmental Studies, Addis Ababa University, Addis Ababa, Ethiopia; 3 Department of Applied Geology, College of Applied Natural Science, Adama Science and Technology University, Adama, Ethiopia; 4 Department of Research Analytics, Saveetha Dental College and Hospitals, Saveetha Institute of Medical and Technical Sciences (SIMATS), Saveetha University, Chennai, Tamil Nadu, India; Wallaga University, ETHIOPIA

## Abstract

Rapid urbanization and the associated expansion of built-up areas are transforming land resources, ecosystem functions, and the spatial structure of cities. Distinguishing urban growth, urban expansion, and urban sprawl is essential for understanding the processes and patterns of urban transformation and supporting sustainable urban planning. This study integrates machine learning and geospatial technologies to analyze spatio-temporal land use and land cover (LULC) dynamics and urban expansion in Gambella Town, Ethiopia. Multi-temporal Landsat satellite images from 2005, 2015, and 2025 were processed using remote sensing techniques and a supervised machine learning approach based on the Random Forest (RF) classifier. Six major LULC classes were identified: Agricultural Land, Bush with Grass, Built-Up Area, Bare Land, Forest Land, and Water Bodies. The classification achieved overall accuracies of 90%, 90%, and 89% for 2005, 2015, and 2025, respectively. The results revealed substantial land transformation and rapid physical urban expansion, with the built-up area increasing from 93 ha (2.0%) in 2005–1,956 ha (41.3%) in 2025. This expansion was accompanied by major transformations of surrounding land resources, particularly the conversion of Bush with Grass and other open areas to built-up land. Agricultural land increased from 634 ha (13.4%) to 1,750 ha (36.9%), whereas Bush with Grass declined from 3,375 ha (71.1%) to 652 ha (13.7%). Socio-economic information collected from 277 respondents indicated that population growth, rural–urban migration, housing demand, employment opportunities, and infrastructure development were major drivers of urban expansion. The study distinguishes urban expansion, represented by the increase in built-up land, from urban sprawl, which refers to the spatial pattern and form of outward urban development. The integration of Random Forest classification and GIS-based spatial analysis provides valuable evidence of land transformation, vegetation loss, and increasing pressure on urban services, supporting more sustainable urban planning and land management in Gambella Town.

## Introduction

Urbanization is one of the most significant demographic and socio-economic transformations of the twenty-first century. Currently, more than half of the global population resides in urban areas, and this proportion is expected to continue increasing in the coming decades. Although urban growth is a worldwide phenomenon, its pace is considerably faster in developing countries, where rapid expansion often exceeds the capacity of infrastructure, governance, and environmental management systems, placing substantial pressure on limited natural and economic resources [1–4].

Urban growth, expansion, and sprawl, characterized by the outward expansion of urban areas into surrounding rural and peri-urban landscapes, has emerged as a major consequence of rapid urbanization [5–9]. This process is primarily driven by population growth, economic development, and rural-to-urban migration. Examining the spatial and temporal characteristics of urban expansion provides valuable insights into the dynamics of urban growth and enables the prediction of future development patterns, thereby supporting sustainable urban planning and resource management. A comprehensive understanding of urban sprawl is therefore essential for developing effective land-use policies, improving infrastructure planning, and promoting environmentally sustainable urban development [10,11].

The continuous expansion of cities has resulted in increasing demand for land, housing, transportation, and public services. As urban areas grow, natural ecosystems and agricultural lands are frequently converted into built-up environments, leading to significant changes in land use and land cover (LULC) [10,12–15]. Monitoring these transformations has become an important component of global environmental change research because LULC changes directly influence ecosystem functions, biodiversity, climate, and sustainable resource management [10,16–18]. Previous studies have shown that uncontrolled urban expansion contributes to the loss of agricultural land, environmental degradation, deterioration of water quality, increased air pollution, fragmentation of green spaces, higher infrastructure and energy costs, and greater socio-spatial segregation [10,11,14,15,19–21].

Urban sprawl generally alters the spatial structure of cities by converting natural and agricultural landscapes into residential, commercial, and industrial developments. Depending on local geographical and socio-economic conditions, urban expansion may occur in concentric forms surrounding established urban centers or linearly along major transportation corridors. Identifying these growth patterns is fundamental for effective urban planning and sustainable land management. Advances in remote sensing (RS), Geographic Information Systems (GIS), and spatial analysis have significantly improved the ability to monitor, quantify, and evaluate urban expansion over time using satellite imagery integrated with ancillary spatial datasets such as topographic maps and field observations [7,8,10,11,22].

Urban sprawl has become a widespread challenge affecting both developed and developing countries. While large-scale suburban expansion was initially associated with the post-World War II development of industrialized nations, contemporary urban growth is increasingly concentrated in developing regions where rapid population increase and migration accelerate the expansion of urban boundaries [3,10]. Such uncontrolled growth often results in environmental degradation, loss of biodiversity, inefficient land utilization, increased demand for public services, and growing pressure on existing infrastructure.

Africa is currently experiencing one of the fastest rates of urbanization worldwide. The proportion of the continent’s urban population increased from approximately 15% in 1940 to about 40% in 2010 and is projected to reach nearly 60% by 2050. Nevertheless, substantial differences exist among African countries. Nations such as Burundi, Ethiopia, Malawi, Burkina Faso, and Uganda continue to have relatively low levels of urbanization, whereas South Africa has experienced much higher urban growth associated with industrialization and economic development [10,15,21,23–25]. The rapid expansion of African cities presents significant challenges for sustainable land management, infrastructure development, and environmental conservation Getu, K., & Bhat, H. G. [2].

Ethiopia is among the least urbanized countries in Sub-Saharan Africa; however, it is currently experiencing one of the region’s fastest urban growth rates Singh Chandel, A. [15]. Urban population expansion has been estimated at approximately 6%, exceeding the average rate for many other Sub-Saharan African countries. Although agriculture remains the dominant sector of the national economy [9–13,16,17,25], increasing rural-to-urban migration, population growth, and economic transformation are accelerating urban expansion. In many Ethiopian cities, residential developments have expanded horizontally through both planned and informal settlements, often extending onto agricultural land and other undeveloped areas Singh Chandel, A. [1,5,6,15]. This pattern of growth has contributed to inefficient land utilization and increasing pressure on urban infrastructure and environmental resources.

Population increase resulting from both natural growth and rural-to-urban migration has been identified as one of the principal drivers of urban sprawl in Ethiopia [2,10,19–21,24–26]. Understanding the spatial and temporal dynamics of urban expansion is therefore essential for supporting evidence-based urban planning and sustainable land management. The novelty of this study lies in integrating machine learning, multi-temporal Landsat observations (2005–2025), GIS-based urban expansion analysis, and socio-economic data to develop a comprehensive framework for assessing urban expansion dynamics in Gambella Town, an understudied and rapidly emerging urban center in Ethiopia. Unlike previous studies focused mainly on major cities or conventional LULC change assessment, this research provides new evidence on urban expansion patterns, land transformation processes, and socio-economic drivers, supporting sustainable urban planning and land management in rapidly expanding secondary cities.

To address this gap, this study integrates Random Forest machine learning, multi-temporal Landsat imagery, GIS-based spatial analysis, and socio-economic data to examine urban expansion dynamics in Gambella Town from 2005 to 2025. The study provides new insights into the magnitude, spatial patterns, and drivers of urban expansion, contributing scientific evidence for sustainable urban planning in understudied secondary cities of Ethiopia.

Therefore, this study aims to analyze the spatio-temporal dynamics of urban expansion in Gambella Town, Western Ethiopia, using an integrated machine learning and geospatial technology approach. Specifically, the objectives of this study are to: (i) classify and quantify land use and land cover changes between 2005, 2015, and 2025 using the Random Forest machine learning algorithm; (ii) evaluate the spatial pattern and magnitude of urban expansion and associated land conversion processes; (iii) identify the major socio-economic factors influencing urban sprawl through household surveys and stakeholder information; and (iv) assess the implications of urban expansion on agricultural land, vegetation resources, and sustainable urban development. The findings are expected to provide valuable scientific information for urban planners, policymakers, and environmental managers to develop sustainable land-use strategies for Gambella Town and other rapidly growing secondary cities in Ethiopia.

## Materials and methods

### Description of the study area

Gambella town, located in the Gambella Region of Ethiopia, has a unique historical background. The area has been inhabited by various indigenous ethnic groups, and others, Ethiopian highlanders who are lives in the region. The Gambella Region is situated in the western part of Ethiopia, bordering South Sudan to the west and south.In the late 19th and early 20th centuries, the Gambella area became part of the Ethiopian Empire. During the Italian occupation of Ethiopia (1936–1941), Gambella experienced some changes in administration and infrastructure [27]

Gambella Peoples’ National Regional State (GPNRS) is located in the southwestern part of Ethiopia, at a distance of 777 km from Addis Ababa. Administratively speaking, the region has three zones and one Special Woreda. There are 226 Kebeles. Five of the kebeles are that of Gambella town. According to the regional government’s policy documents, GPNRS is one of the emerging regions of the country and a home for the five indigenous ethnic groups, the Nuer, Anywaa, Majang, Opo, and Komo. In addition, following the resettlement program of the 1980s, a good number of people from different parts of the country moved to the region. Recently, the refugees from South Sudan (estimated 350,000) are becoming the most dominant, imposing a threat to the environment, socioeconomic and cultural values. Gambella is located in Ethiopia with 8.12, 50 N- 8.16, 20N and 34.33, 00 E −34.37, 40 E coordinates (Fig 1). It shares a long border with South Sudan and two other Ethiopian regions: Oromia to the north and east and the Southern Nations, Nationalities and Peoples’ Regional State (SNNPRS) to the south.

**Fig 1:**
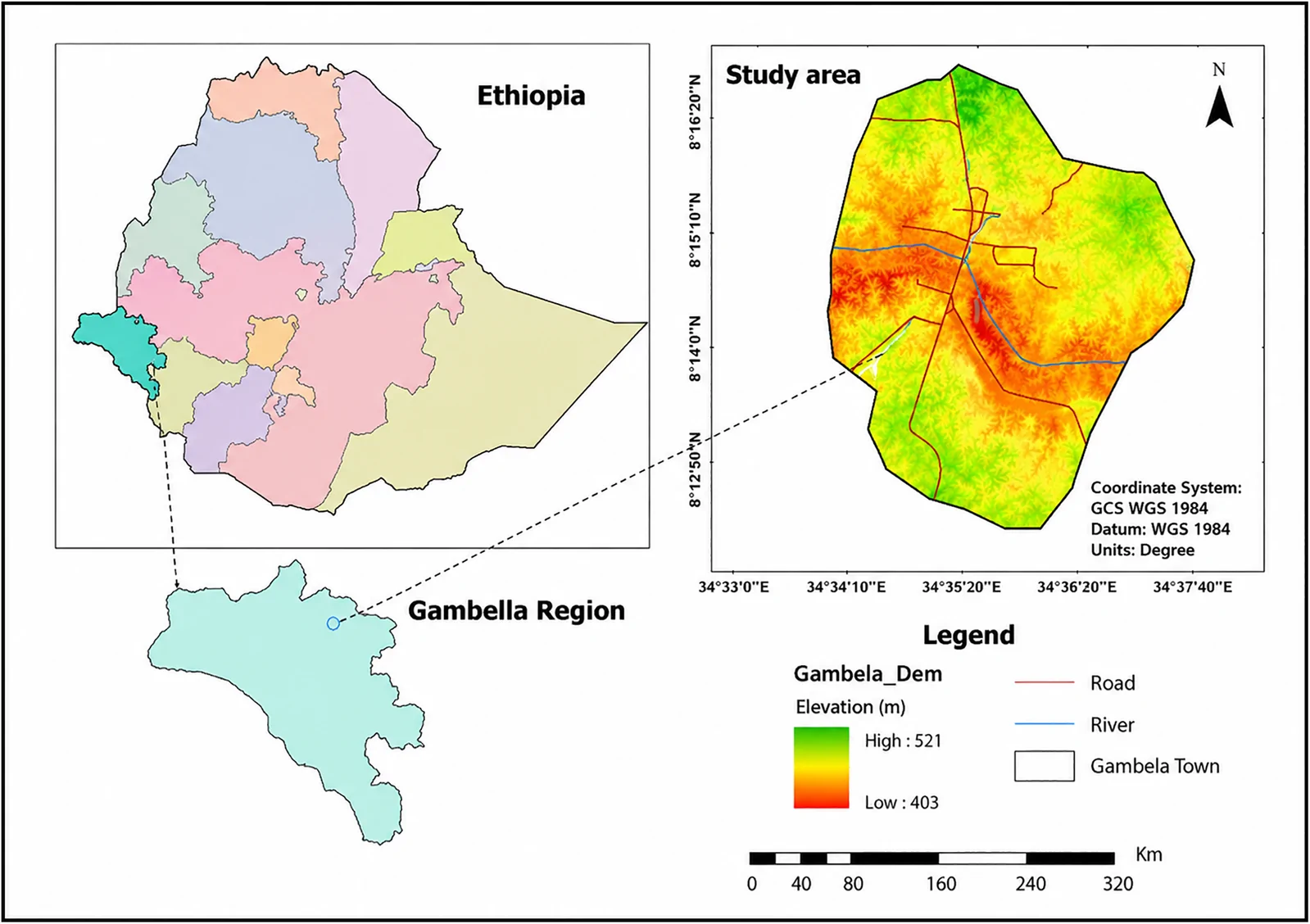
Location of Gambella Town, Ethiopia. Map prepared by the authors using publicly available geospatial and administrative boundary data. This is an original author-generated figure and was not reproduced or adapted from a previously published figure.

### Population

According to the official estimates from the Ethiopian Statistics Service, the projected population of Gambella town in 2017 was 75,098. In the 2017 projected population included 35,802 women, making up approximately 47.7% of the total, and 39,296 men, accounting for about 52.3%. This gender distribution indicates a slightly higher number of males compared to females. The detailed demographic information can be further explored in the accompanying figure (Fig 2), which illustrates the population dynamics and proportions within the town.

**Fig 2:**
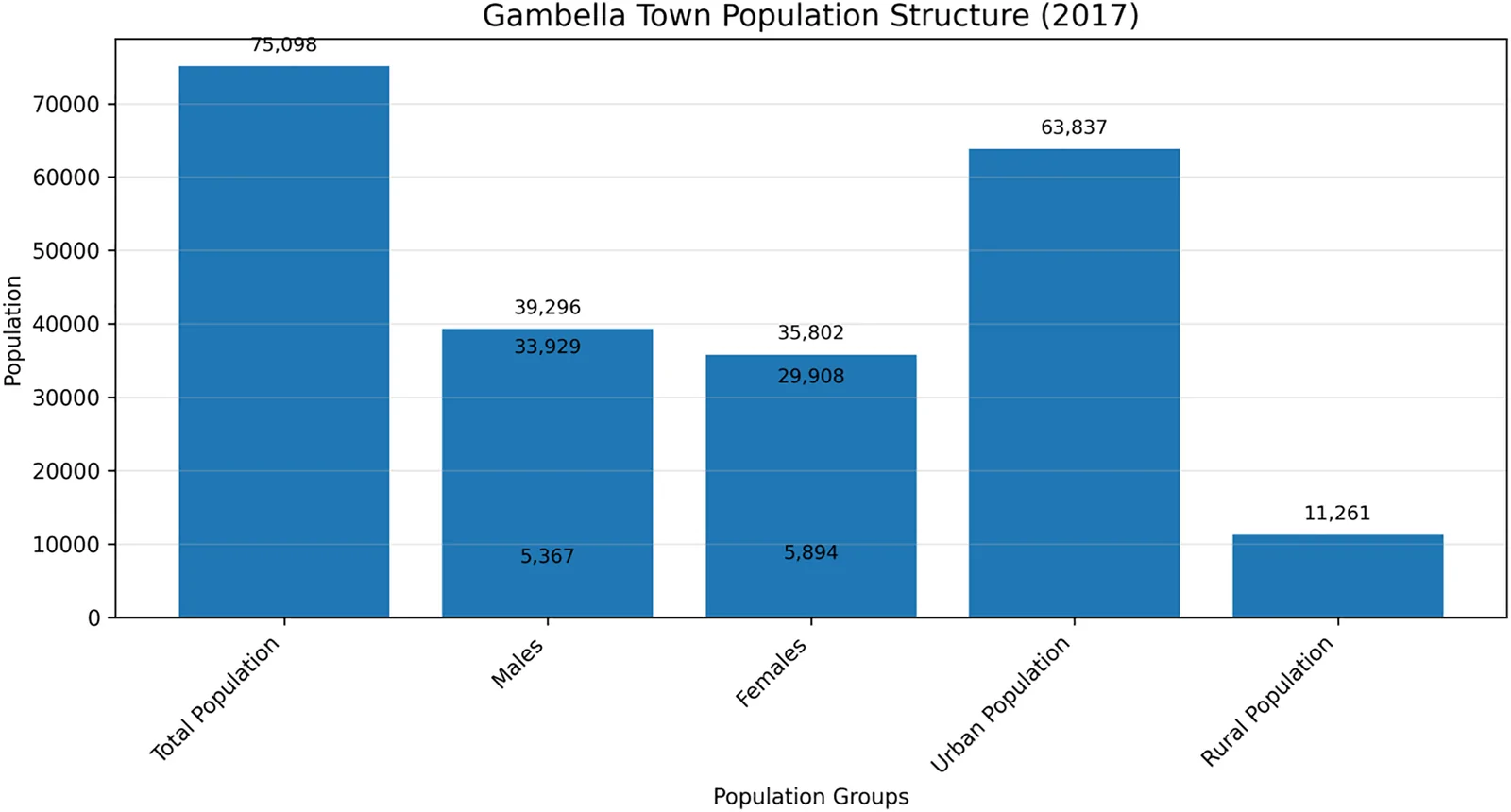
Population of the study area.

### Software and instruments

The GIS software and Machine learning utilized in this investigation was widely known and used for image processing. A GPS device is used in order to gather ground truths for field verification. Image processing was also accomplished with the aid of Google earth engine. The structured questionnaire was initially written in English and then translated into Amharic. The questionnaire is then given out to the sample of expert and the community. Full details are displayed in the Table 1.

**Table 1:** Description of the software and their purposes.

No.	Software Version	Purpose
1	ArcGIS 10.8	To do tasks such as cutting, Georeferencing for cross-tabulation (calculating the LULC class matrix between photos taken at various times), creating a map layout, extracting polygons from raster’s, and creating slope and elevation maps using SRTM data
2	Machine learning(RF)	For layer stacking, sub-seting, clipping, classifying the various LULC categories, post-classification, computing area, and evaluating the classification accuracy.
3	Machine learning (Jupytor)	Is a powerful software suite used for statistical analysis, data management, and data documentation
4	Garmin 72 GPS/ Essentials GPS	To gather the control point for ground truth.
5	Digital Camera	To take pictures of the research area for photography

### Sample size and sampling techniques

The sample size was calculated in a Jupyter Notebook environment using Python based on the simplified formula proposed by Kasunic (2005):


$$\begin{array}{c} \text{n}=\frac{N}{\text{1}+N{\left(e\right)}^{\text{2}}} \end{array}$$

where n represents the required sample size, N represents the total population size (75,098), and e represents the desired precision level (0.06). The Python-based calculation resulted in a sample size of 276.62, which was rounded to 277 respondents. This sample was used for machine learning model training, testing, and accuracy evaluation.

### Ethics and informed consent

The study involved the collection of non-clinical socioeconomic and perceptual information through structured questionnaires and consultations with experts, local specialists, and community members. Participation was voluntary, and informed verbal consent was obtained from all participants before data collection. Before participation, the purpose of the study, the nature of the information requested, the intended academic use of the information, and the voluntary nature of participation were explained to each participant. Participants were informed that they could decline to answer any question or withdraw from the discussion at any time without consequence. Because the information collected was non-sensitive and no personally identifiable information was recorded, verbal consent was considered appropriate. The verbal consent was documented by the research team in the field records, and consent was witnessed by members of the research team. No names or other directly identifying information were included in the research dataset or manuscript.

### Digital image processing

Digital image processing focuses on improving the usability of distorted or inferior image data for accurate interpretation and representation of scenes [25,28–31]. This involves preprocessing raw image data to eliminate noise, calibrate radiometric information, and correct geometric distortions. Techniques such as contrast stretching and histogram equalization enhance visual quality, making images clearer for human observers and more effective for automated analysis. Image classification, particularly through methods like maximum likelihood classification (MLC), sorts pixels into land cover categories based on their reflectance characteristics, facilitating detailed analysis of land use and environmental changes Getu, K., & Bhat, H. G. [2,32–36].

Supervised classification further refines this process by using training samples to categorize pixels into distinct land use/land cover (LULC) classes Getu, K., & Bhat, H. G. [2]. Ground truthing activities validate these classifications by collecting GPS data and verifying various terrain types in the study area. This comprehensive approach ensures the reliability of the data used for classification, ultimately enhancing the accuracy of information derived from remote sensing. By integrating these methodologies, digital image processing plays a crucial role in applications such as environmental monitoring, land-use planning, and resource management.

### Data description and data source

The study has used Landsat satellites imageries of 2005, 2015 and 2025 was the main data source for the dependent variable for spatial temporal change of urban expansion pattern. Is a freely available satellite image downloaded from the USGS database (https://earthexplorer.usgs.gov/), all data are supplemented with secondary data sources. Full details are displayed in Table 2 below.

**Table 2:** Description of the description and data sources.

GIS Data layer	Data Description	Data source
Vector (polygon)	Woreda Boundary	Ethio-GIS
Vector (line)	River, Contour and Roads	Ethio- GIS
Attribute table	Population	CSS,2017
Vector (point)	Major Town	Ethio-GIS
Raster	Elevation	DEM 12.5m from SSGI
Raster	Satellite Image 2005 Landsat4 TM 2015 Landsat7ETM+ 2025 Landsat 8 OLI & TIRs	USGS 2005,2015 and 2025 (Image courtesy of the U.S. Geological Survey)
Ground truth	Google Earth pro	Google Earth pro

Data from many sources have been gathered for the study. First, structured questionnaires are used to analyze the effects of urban expansion on the study area; digital cameras are used to take photos of the sample filed area; GPS is used to obtain ground truth points for accuracy assessments; field observations are conducted; and data obtained from other sources is substantiated by questioners with experts and affected individuals. The history and current state of the city’s LULC are also discussed with local specialists and elders who work for the municipality.

The Space science and Geo-spatial Institute (SSGI) is one of the offices from which secondary data is gathered. A variety of published and unpublished sources, including books, journals, the internet, research reports, articles, documents studied for this investigation, and other materials from the Gambella Land administrative and urban development plan provided additional supplementary data for this study.

### Remote sensing data acquisition and methods of data collection

Most of the data for this study comes from satellites, both primary and secondary data sources were used to achieve the intended objectives. Among the well-known open-source satellite imagery, Landsat satellite imageries are ideal for this purpose of analysing the spatial and temporal changes of urban expansion pattern with good spatial resolution. Therefore Landsat 4–5 TM, Landsat 7 ETM+ and Landsat 8–9 OLI_TIRS imageries was used for this study to assess the spatial temporal change of expansion pattern over the period considered when downloaded from the USGS database. Others data were used to collected from the Ethiopia Geospatial institutes. Full details are displayed in Table 3 below.

**Table 3:** Description of the metadata of satellite images.

№	Data type	Raw and Path	Acquisition Date	Spatial Resolution	Data Source
1	LANDSAT_4–5 TM	171/054	2005-02-02	30m x 30m	USGS Earth Explorer
2	LANDSAT_7ETM+	171/054	2015-01-04	30m x 30m	USGS Earth Explorer
3	LANDSAT_8–9 OLI_TIRS	171/054	2025-01-08	30m x 30m	USGS Earth Explorer

The Land use and land cover changes (LU/LCC) in Gambella town between 2005 and 2025 the area of interest was extracted from the larger scene of, Landsat 4–5 TM, Landsat 7 ETM+ and Landsat 8–9 OLI_TIRS imageries using the. Machine Learning Classification techniques were used to employed the categorize of images into different land cover types.

Generally, the Earth Explorer website (USGS) provided Landsat images for 2005, 2015, and 2025, while https://libra.developmentseed.org provided Landsat 8 imagery. These sources served as the first step in the study technique. The Fig 3 illustrates a systematic and integrated approach for analyzing urban expansion and land use/land cover (LULC) dynamics. The study begins with data collection and acquisition, drawing from primary sources such as GPS ground truth, questionnaires, field observations, and digital camera records, alongside secondary datasets including population statistics, Ethio-GIS data, Addis Ababa University, SSGI reports, and literature, as well as satellite imagery (Landsat TM 2005, ETM + 2015, OLI/TIRS 2025) and DEM data. These datasets undergo rigorous pre-processing steps, including image download, layer stacking, clipping, enhancement, and geometric and radiometric corrections to ensure data quality. A Random Forest machine learning classifier is then applied for land use/land cover classification, followed by accuracy assessment using ground truth data, confusion matrix, overall accuracy, and kappa coefficient. Subsequent stages involve LULC change detection (2005–2025), spatial analysis of urban expansion (including area change, population, slope, and elevation factors), and statistical analysis of questionnaire data using Python/Jupyter tools. The outputs are then synthesized into maps, tables, and graphs for visualization, culminating in interpretation of results to support conclusions on urban expansion dynamics and their driving factors.

**Fig 3:**
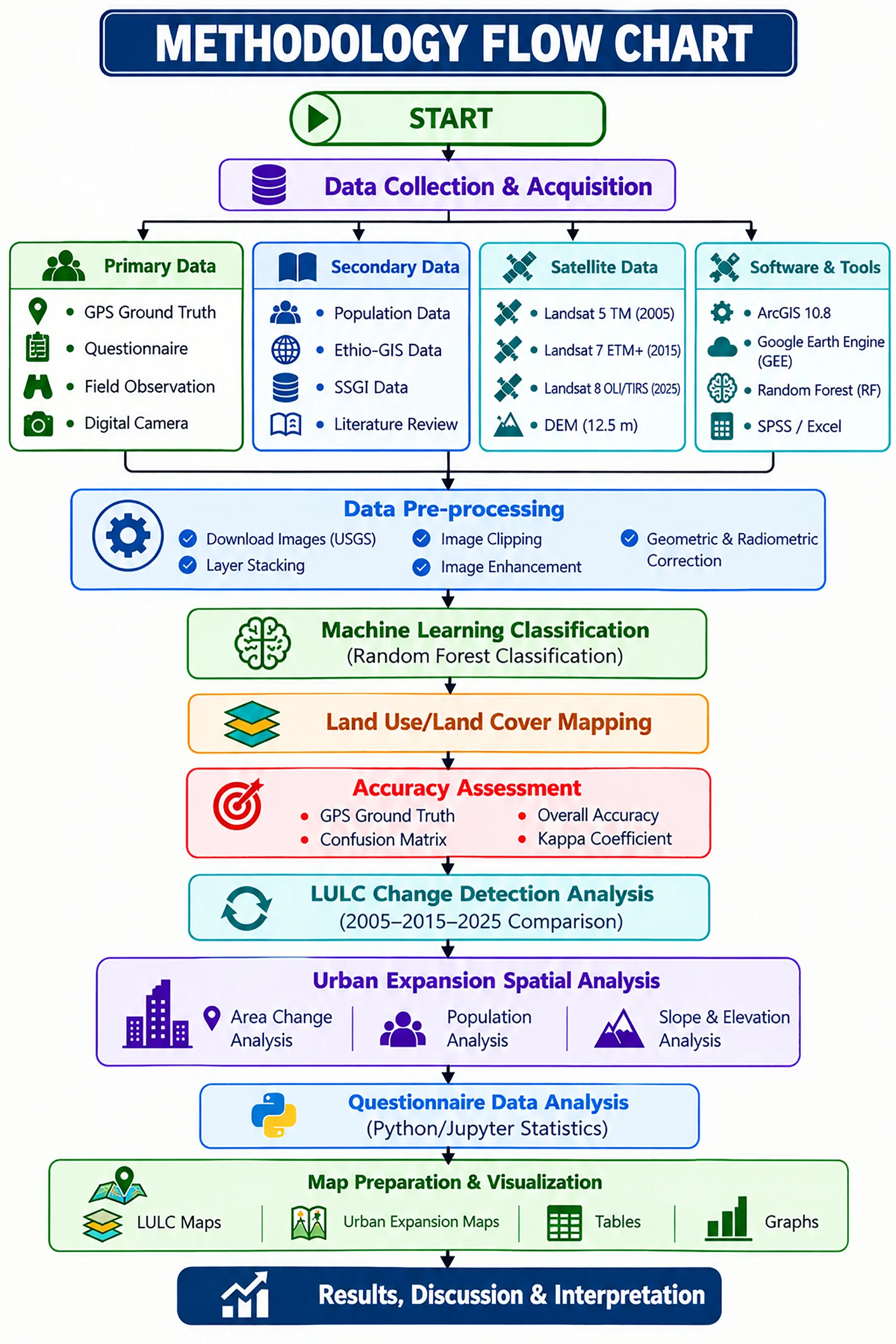
Methodology flow chart frameworks.

### Random Forest classification and accuracy assessment

A supervised Random Forest (RF) classifier was used to classify the Landsat imagery into the identified LULC classes for 2005, 2015, and 2025. Training samples were selected based on field observations, GPS ground-truth data, Google Earth imagery, and visual interpretation of the satellite images. A total of 200 ground-truth points were collected and distributed across the identified LULC classes to represent the spatial and spectral variability of the study area. Care was taken to select representative and relatively homogeneous locations for each class.

The collected reference data were used to support the training and validation of the RF classification. The RF algorithm was applied to the preprocessed Landsat spectral bands to generate the LULC maps for each study year. The same LULC classification scheme was maintained across the three study periods to ensure consistency in the subsequent change-detection analysis.

The accuracy of the classified LULC maps was assessed using a confusion matrix, overall accuracy, and Kappa coefficient. The classified pixels were compared with the corresponding reference/ground-truth points to determine the number of correctly and incorrectly classified samples for each LULC class. Overall accuracy was calculated from the proportion of correctly classified reference samples to the total number of reference samples, while the Kappa coefficient was used to measure the agreement between the classified map and reference data beyond chance agreement. Accuracy assessment was conducted for each study year to evaluate the reliability of the resulting LULC maps.

**Ethics Statement: Not applicable. This study did not involve human participants or human subjects.**

## Results and discussions

### The socioeconomic characteristics

#### Character of respondents.

The Respondents’ demographic information, including sex, age, marital status, education, occupation, and family size, was gathered because socio demographic traits of a population have an impact on the issue of urban expansion. The distribution of family chores and decision-making is significantly impacted by the sex makeup of the population, which in turn affects the community’s standard of living. With men making up 56% of the total and women 44%, male respondents were generally found to be more numerous than female respondents. This is due to the fact that male partners often make the majority of decisions about landholding and land use.

The respondent’s age distribution is important to determine which age group is most at risk of losing their means of subsistence. According to the data, most of the respondents are in the working or operating age range. The sample’s adult age group comprises around 61% of the total respondents (20–40 years old). Of the 277 respondents, 169 (61%) were between the ages of 20 and 40, 65 (about 23%) were between the ages of 41 and 60, and 43 (16%) were over the age of 60, according to the study. Generally speaking, the age group structure shows a significant percentage of the respondents were discovered to be between the ages of 20 and 40, which is reproductive age and suggests the possibility of population expansion, one of the factors contributing to urban expansion, which is the unplanned spread of cities or towns as a result of an expansion of the population and migration.

Most of the town’s residents have a high level of education. The educational attainment of 114 (41%) of the respondents who are educated is shown in the table below, they have a first degree or above of the responders, roughly 21 (8%) finished primary school, while roughly 54 (19%) finished secondary school. About 34 (12%) and 46 (17%) of the population have certificates and diplomas, respectively, while the remainder 8 (3%) are illiterate. With the assistance of the researcher or data collector, these 85 respondents completed the questionnaire.

Family plays a key role in social adjustment, economic standing, and income maintenance.

Statistics on marriages therefore have socioeconomic ramifications 87 (31%) of the respondents were single or never married, 35 (13%) were divorced, and 12 (4%), respectively, were widowed, whereas 143 (52%) were married (Fig 4). As a result, the respondents’ responses might draw from their personal experiences managed caregiving and family responsibilities.

**Fig 4:**
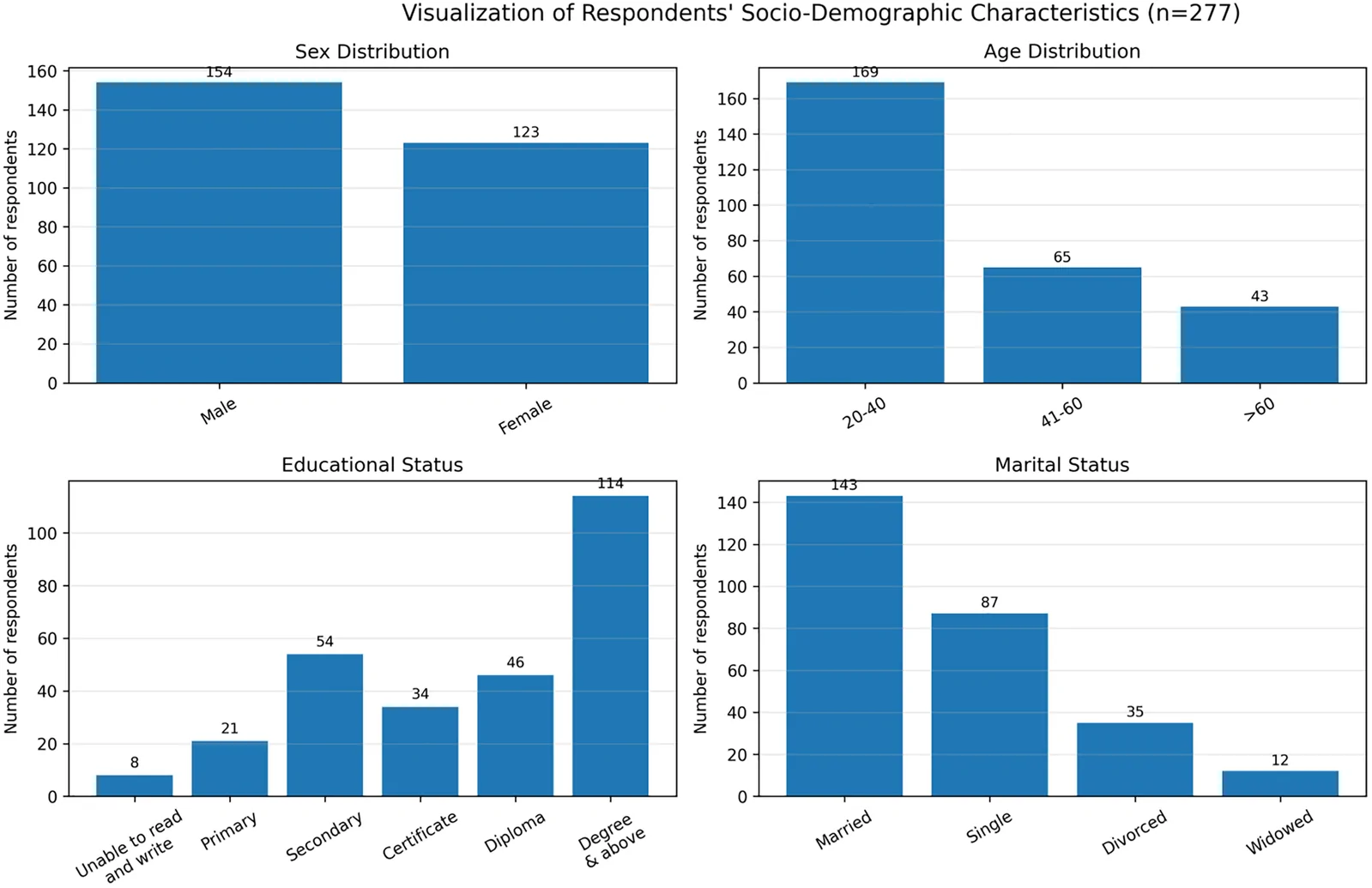
Socio demographic visualizations.

#### Family size.

The typical size of households in the research region, the largest household size was found to be 142 (5–10) members; the second largest was 75 (0–5) persons; and the third was 60 (>10) or more than nine members. In general, the figure indicates that the majority of sample homes had large families, with the average respondents’ family size being between five and ten members. It is employed as a gauge of population crudeness and has significant effects on how urban sprawl affects their means of subsistence. The majority of suburbanites struggle to provide for their families with the resources available to them.

#### Household occupational status.

The occupational status distribution indicates that civil servants constitute the largest proportion of respondents (32%), followed by daily workers (21%), unemployed individuals (15%), farmers (13%), and traders (11%), while the private sector (6%) and other occupations (2%) represent relatively small shares (Fig 5). This distribution suggests that the study area has a strong dependence on public-sector employment, reflecting the role of government institutions as a major source of livelihoods. The relatively high proportion of daily workers and unemployed respondents also points to limited formal employment opportunities and highlights potential socio-economic challenges, including income instability and labor market vulnerability. The lower representation of private-sector employment indicates a relatively weak private economy compared to the public sector. Overall, the occupational structure reflects a mixed urban economy dominated by government employment, with a considerable share of informal and insecure occupations, emphasizing the need for policies that promote private-sector investment, entrepreneurship, and job creation to enhance sustainable economic development.

**Fig 5:**
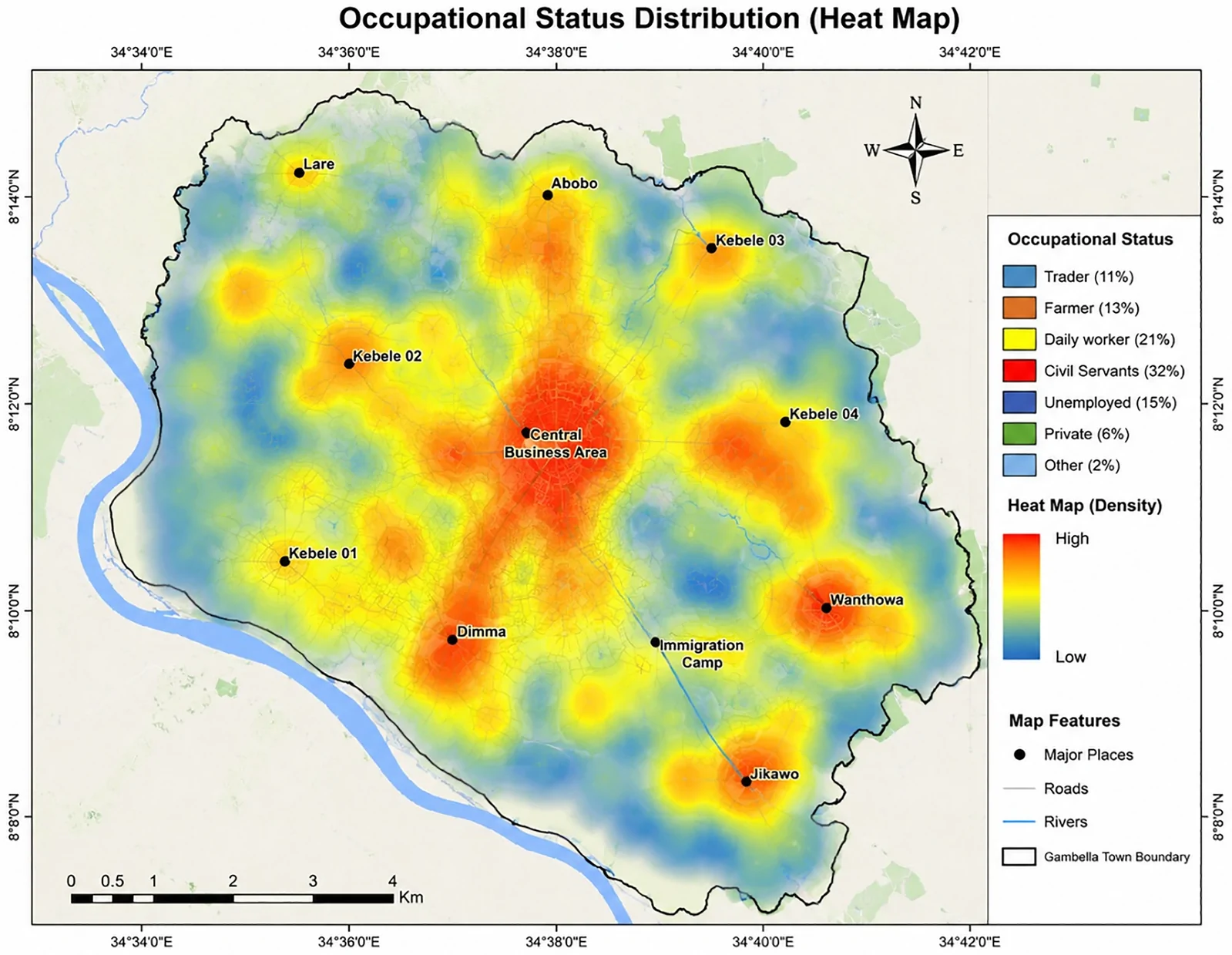
Household occupational status of respondents in Gambella Town. Figure prepared by the authors based on household survey data collected for this study. This is an original author-generated figure and was not reproduced or adapted from a previously published figure.

#### Monthly income of households.

The socioeconomic element of income is another reason why people leave their place of origin, and they are looking for work, which makes it challenging to forecast the rate of local population growth. For a variety of factors, including poor educational attainment, looking for batter job, health services and infrastructures likes roads, which makes people dread taxes, particularly traders, the difficulty to maintain sales records, and the fact that most had inconsistent and changeable income, it was exceedingly difficult to find information on the household heads’ income. The numbers of respondents, however, have monthly incomes which are greater than 5000 birr (35%) and between 2001 and 5000 birr (23%). The monthly incomes of 18.0% and 12.0% of respondents, respectively, are between 1501 and 2000 and 1001 and 1500 birr. However, the remaining 9% are between 501 and 1000 birr and less than 3% have 500 birr a monthly, respectively this data was analysis from collected data from respondents (Fig 6).

**Fig 6:**
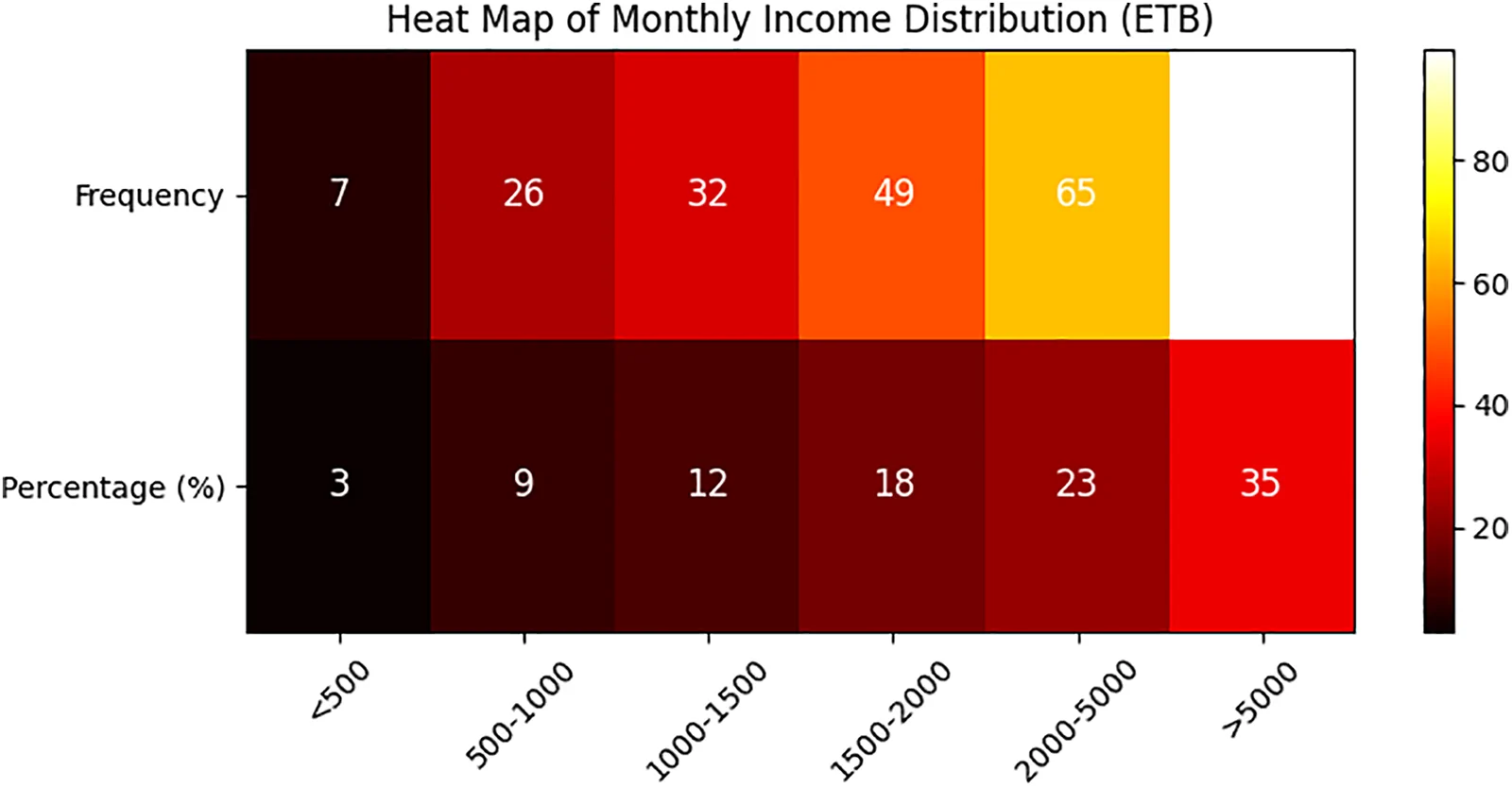
Description of the monthly income.

#### The status length of residence in the Gambella town.

According to the respondents’ length of residency in the Gambella town, 73% of the respondents are indigenous citizens and were born in Gambella regions. The remaining 26%, however, were immigrants, particularly from Ethiopia highlanders, South Sudanese refugee who seeks for slum and the surrounding region. One of the signs of the urban sprawl issue is the high rate of migration in the town, as seen in Fig 7.

**Fig 7:**
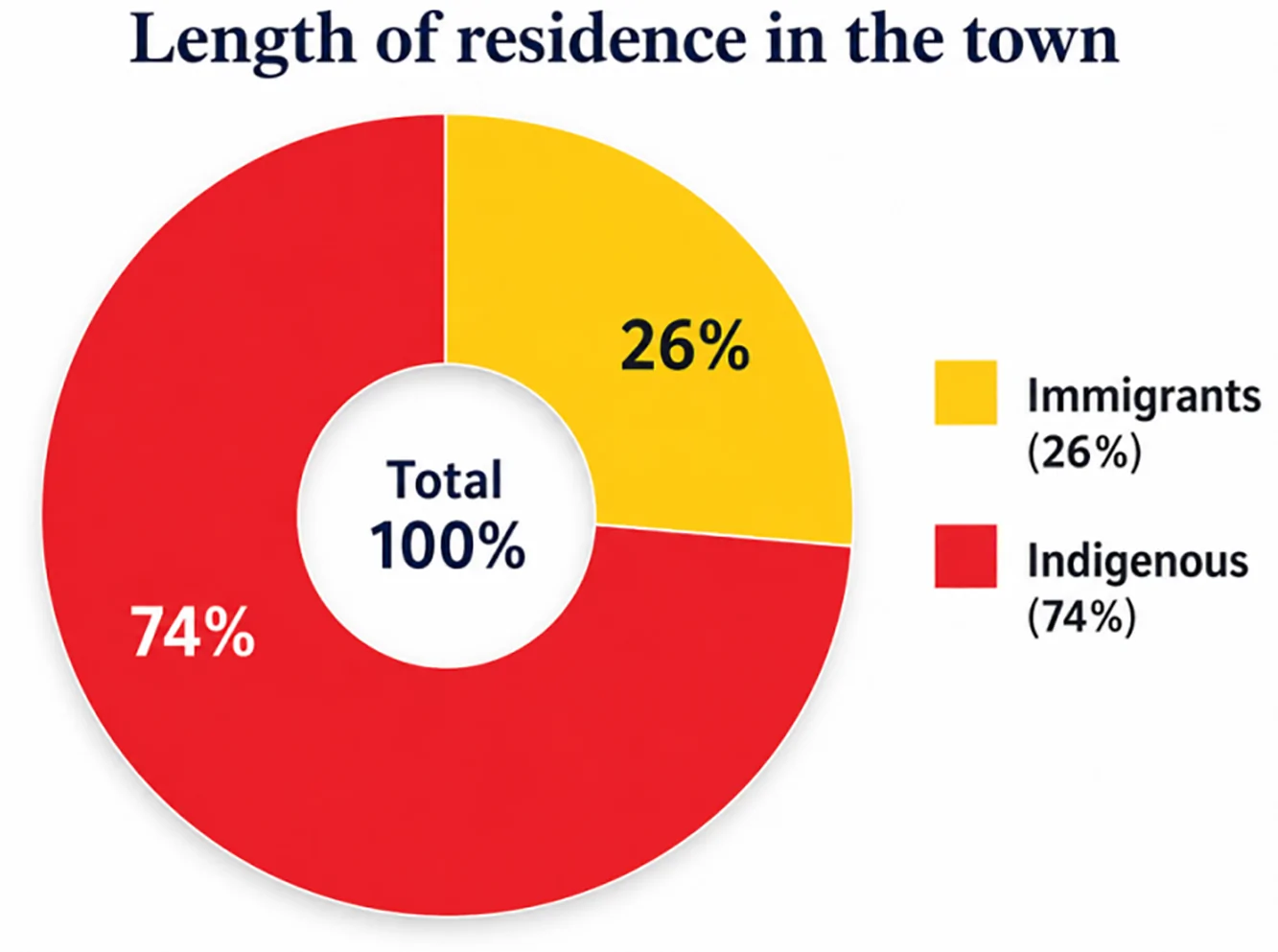
Length of residence in the town.

### The socioeconomic characteristics of the respondents’ household heads

#### Land ownership status.

About 67% of all respondents own land, whereas the remaining 33% have no land due to the lack of urban settlement policy, and poor management of land use in the study area.

That the majority of respondents (161, or 58%) do not own any land in hectares, while 35, or 13%, have land in the hectares. The remaining 81, or 29%, of the respondents own land in care.

#### Land use categories.

The respondents are also using their land for a variety of purposes, including housing, grazing, cultivating crops along the Baro river bank, and mixed uses. Crop land accounts for 16% of their total land, followed by pasture land, fruit and vegetable land, and crop land combined. Housing and grazing land make up 39% and 12% of their amount, respectively (Fig 8). Consequently, the farmers’ land contributes to their income or means of subsistence. This suggests that their source of income is directly impacted by losing the land they own, unless it is replaced by another source of income.

**Fig 8:**
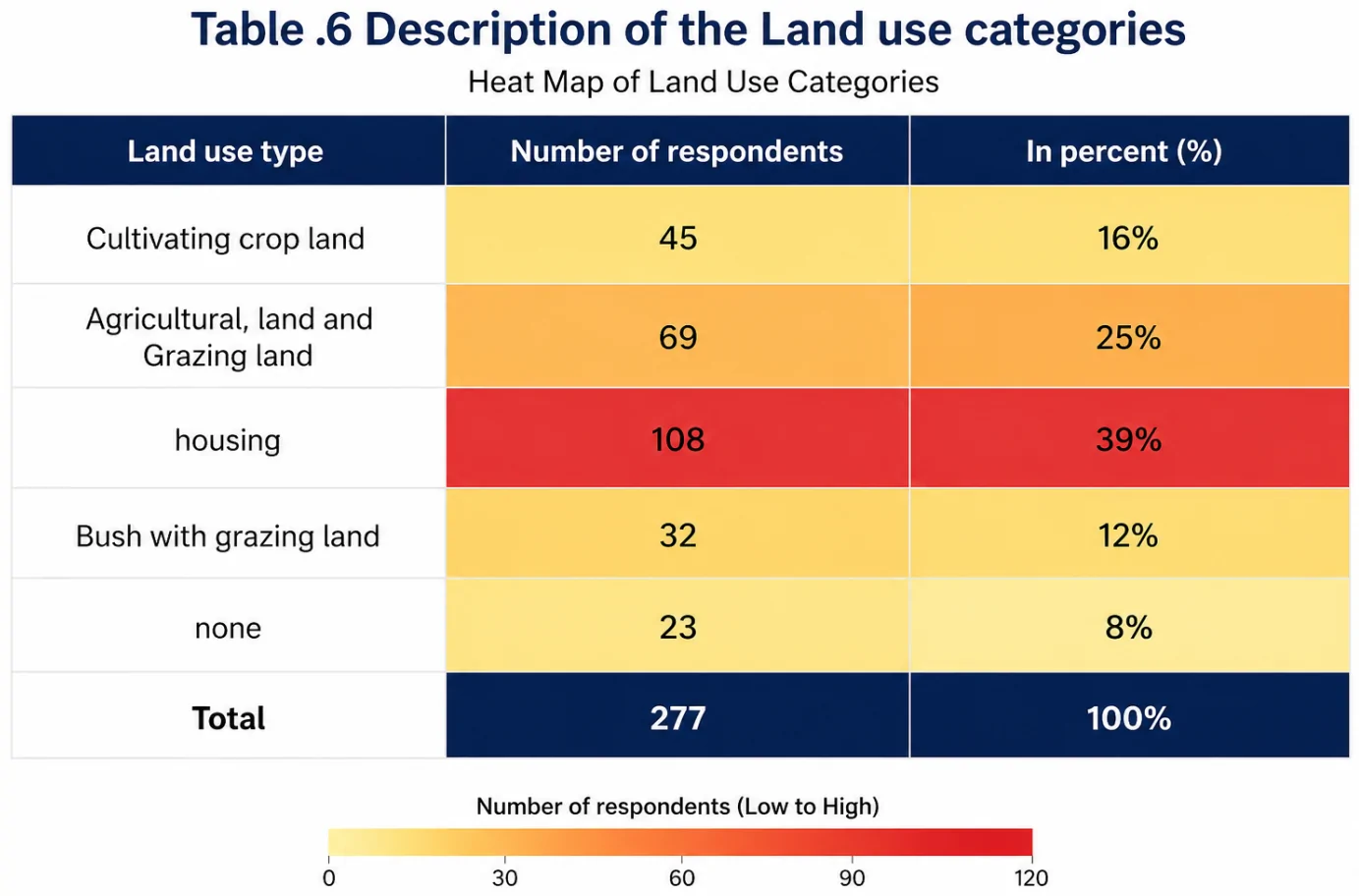
Descriptions of the Land use categories.

### Urban expansion of Gambella town

#### Type of change observed in Gambella town.

As shown in Fig 9 below, the majority of respondents (34%) respond that the expansion of built-up area and the replacement of agricultural land in built-up areas. This accounted for 34% of the population increase, followed by the expansion of built-up land, infrastructure, and investment in agricultural areas to built-up areas, which accounted for 25% of the population increase. The expansion of built-up land accounts for 13% and 10% of both population growth and investment, respectively, according to 18% of respondents who noted the conversion of agricultural land to built-up area. Therefore, it is feasible to draw the conclusion that the primary shift affecting the lives of farmers who depend on their land and the nation whose economic activity is heavily reliant on agriculture is the conversion of agricultural land to built-up space. The second that is most commonly described is population increase, which contributes to low employment opportunities for town residents and is a major factor in sprawl.

**Fig 9:**
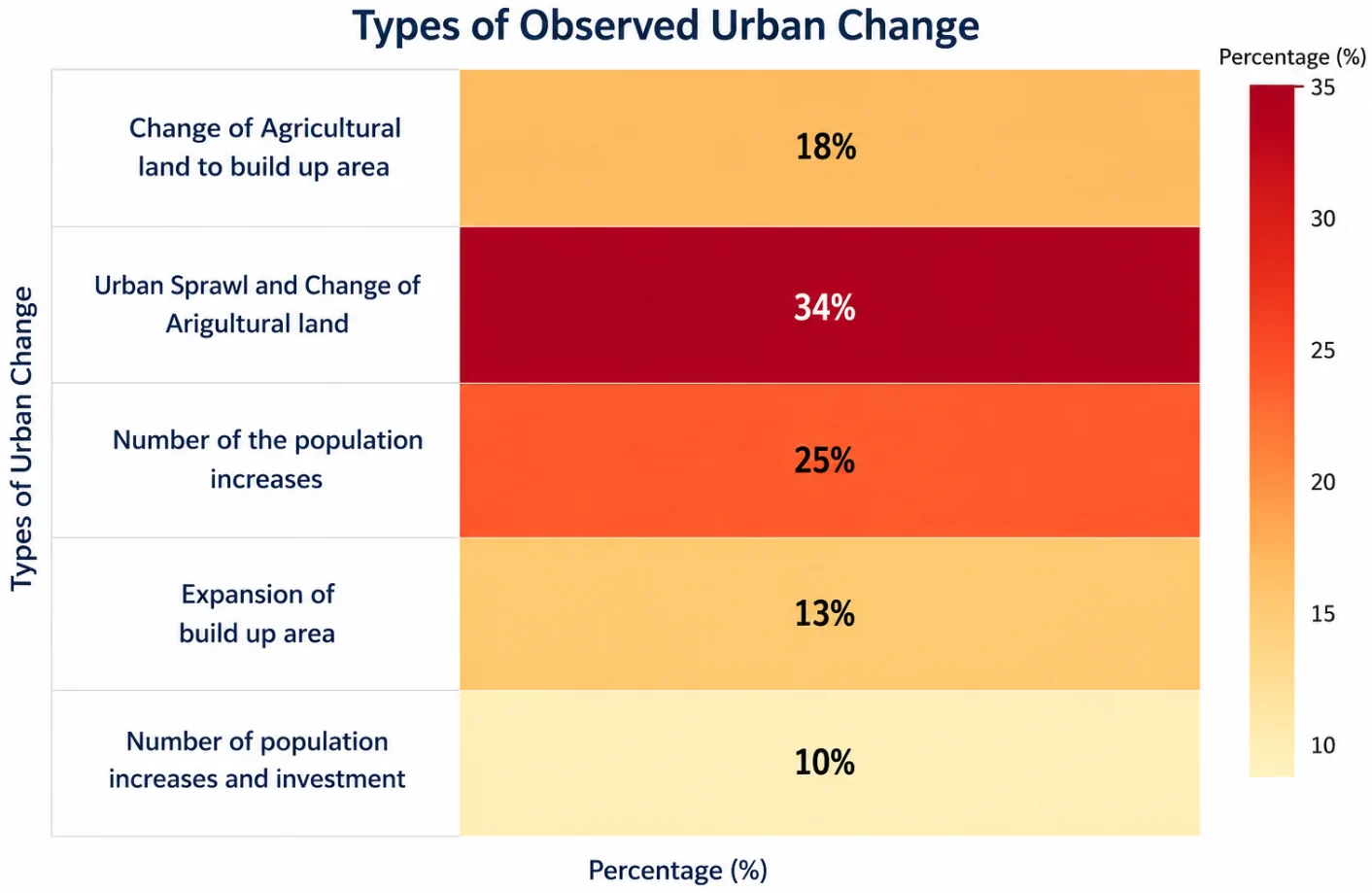
Type of observed urban change.

#### Factors of expansion.

Migration from rural area to urban area, to seek for batter services and population increase together are the main factors contributing to Gambella town’s with 69% growth, as seen in Fig 10 The combined percentage of low land costs, migration, population growth, infrastructure, and land sales is 69%. Forty eighty percent is taken via migration. Migration and cheap land have a combined 14%, whereas 7% of respondents claim that infrastructure and land sales make up 10%. Towns, that determine rapid population growth and migration become unplanned, which leads to illegal home construction (houses without plans) and leapfrogging development (Fig 10).

**Fig 10:**
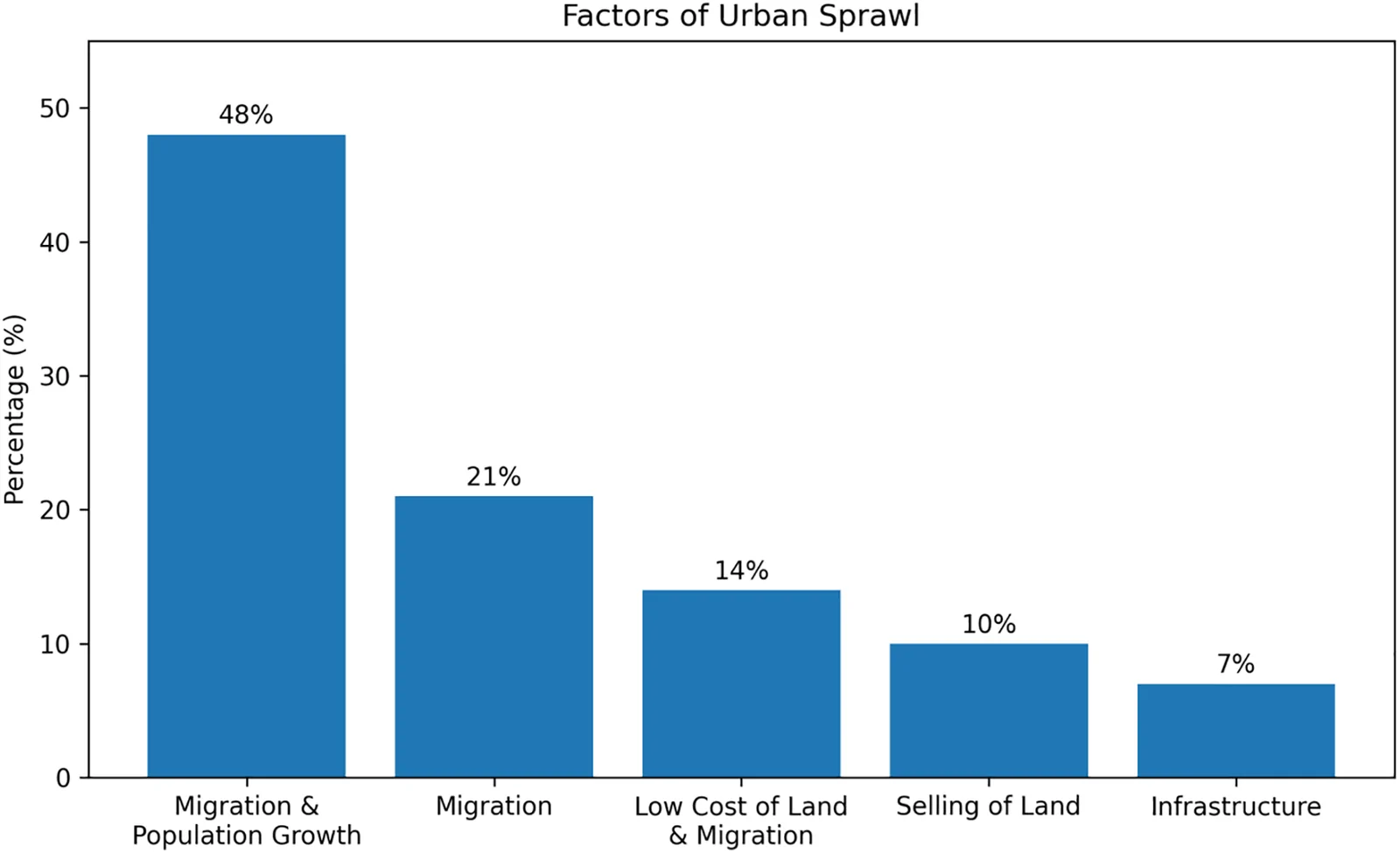
Factor of urban expansion. Source: The data from field survey (2025).

#### Impact of urban expansion in Gambella town.

Table 4 Shows, that 68% of the land taken both from bush with grass and farmers has been used for residential use (6%) is used for agriculture, housing, and grazing, while the remaining portion has been used for grazing and/or one or more of the aforementioned uses. Farmers’ livelihoods are thus immediately impacted by urban expansion’s impact on nearby farmland since it alters their farming practices, within the Baro Rivers Bank and the fertility of soil due to gentle slope which are impossible without land, and the town’s land usage and cover and the environment impact on the loss of biodiversity spaces and wild animals.

**Table 4:** Description of the vacates type of land.

Type of land uses	Number of respondents	In Percentage
Land used for agriculture	28	10
Land for residential use	188	68
Bush with grazing land	23	8
Land used for agriculture, housing, and grazing	16	6
Land used for agriculture and grazing	12	4
Both residential and agricultural property used for grazing	10	4
Total	**277**	**100**

#### The effects of urban expansion.

About 98% of the respondents said that the town’s growth and selecting of new site due to the urban expansion was having an impact on their lives which can cause the ethnic valances, resources competition and power struggle among indigenous citizens, when asked if sprawl affected their lives, as shows in Table 5. The town’s expansion isn’t hurting their lives, according to just 2% of respondents.

**Table 5:** Description of the responses of the participants to the impact of urban expansion.

Recognizing the impacts of urban sprawl	Number of respondents	In percent
Yes	272	98
No	6	2
Total	**277**	**100**

Source: The data collected from a field survey.

The majority of respondents, work for environmental management and land administration bureaus, but some are employed by municipal bureaus. The majority of the answers provided by experts are somewhat standard. They think that distinct land use and land cover classes are changing, and that the amount of built-up land is increasing significantly from 2005 up to 2025 by an estimated 40.2% for various purposes while the amount of agricultural increasing and bush with grasses and forest land is decreasing. People’s need for fixed assets (land), the necessity for a market place, industry, and bad land management, which leads to an increase of unlawful buildings, is the main causes of this transformation. It is well acknowledged that the displacement of local residents or farmers due to changes in land cover has an effect on the lifestyles of urban dwellers. Due to the land’s difficulty, the town’s management is forced to focus on this matter rather than other matters. Natural resource depletion significantly reduces the output of crops like maize, beans and cotton and also leads to many environmental issues like pollution. Ultimately, the proposed solution to the issue caused by the growth of the built-up area is raising awareness, properly implementing the master plan (creating good governance), enforcing the law and regulations, and fostering collaboration among the community, town administration, and the relevant body of government.

### Statistical analysis of socioeconomic drivers of urban expansion

A binary logistic regression analysis was conducted to statistically evaluate the socioeconomic factors associated with urban sprawl. The model included migration, population growth, infrastructure development, low land cost, and land sales as explanatory variables. The overall regression model was statistically significant (LR χ² = 44.524, p < 0.001), with a McFadden pseudo-R² of 0.120, indicating that the included socioeconomic factors provided explanatory power for the occurrence of urban expansion.

As shown in Table 6, migration was significantly and positively associated with urban sprawl (B = 1.191, p < 0.001; OR = 3.289). This indicates that respondents identifying migration as a contributing factor had higher odds of reporting urban-sprawl conditions. Population growth was also statistically significant (B = 0.736, p = 0.007; OR = 2.087), indicating a positive association with urban expansion. Similarly, low land cost showed a significant positive association (B = 0.825, p = 0.006; OR = 2.281), while land sales were also significantly associated with urban sprawl (B = 0.972, p = 0.003; OR = 2.643).

**Table 6:** Binary logistic regression analysis of socioeconomic drivers of urban expansion.

Socioeconomic factor	B	SE	Wald	*p*-value	OR	95% CI
Migration	1.191	0.272	19.108	<0.001	3.289	1.929–5.610
Population growth	0.736	0.275	7.156	0.007	2.087	1.217–3.577
Infrastructure development	0.512	0.285	3.226	0.072	1.669	0.954–2.918
Low land cost	0.825	0.297	7.700	0.006	2.281	1.274–4.083
Land sales	0.972	0.326	8.903	0.003	2.643	1.396–5.005

**Model statistics:** N = 277; LR χ² = 44.524, *p* < 0.001; McFadden pseudo-R² = 0.120.

In contrast, infrastructure development showed a positive but statistically non-significant association with urban expansion (B = 0.512, p = 0.072; OR = 1.669). Therefore, based on the regression results, migration, population growth, low land cost, and land sales were the statistically significant socioeconomic factors, whereas infrastructure development did not reach the 5% significance level.

### Land covers types and its coverage in 2005 and 2025

From the classified image of 2005, the percentage coverage of Bush with Grass (Bush land mixed with grassland) is higher it covers 71.1% of the area. The LULC classification for 2005 from the TM satellite image (Table 7 and Fig 11) showed that Agricultural land makes up 13.4% (634 ha.), bare land covers 9.8% (466 ha.), Built-Up area covers 2% (93 ha.), Water Body (river and ponds) covers 1.7% (83 ha), and Forest Land holds 2% (93 ha), of the district in 2005. Bush with Grass and Agricultural land are the dominant cover types in the district, while water bodies, Forest land and Built-Up area accounted for a very small percentage in 2005

**Table 7:** Descriptions of the land cover types and its coverage in 2005-2025.

ID	2005 Class Name	Area_Ha	Area %
1	Water Body	83	1.7
2	Forest Land	93	2.0
3	Agricultural Land	634	13.4
4	Bush with Grass	3,375	71.1
5	Built-Up area	93	2.0
6	Bare Land	466	9.8
	**Total**	**4,744**	**100**
ID	**2015 Class Name**	**Area_Ha**	**Area_%**
1	Water Body	77	1.6
2	Forest Land	81	1.7
3	Agricultural Land	754	15.9
4	Bush with Grass	3,053	64.4
5	Built-Up area	456	9.6
6	Bare Land	323	6.8
	Total	4,744	100
No	**2025 Class Name**	**Area_Ha**	**Area_%**
1	Water Body	82	1.7
2	Forest Land	70	1.5
3	Agricultural Land	1,750	36.9
4	Bush with Grass	652	13.7
5	Built-Up area	1,956	41.3
6	Bare Land	234	4.9
	**Total**	**4,744**	**100**

**Fig 11:**
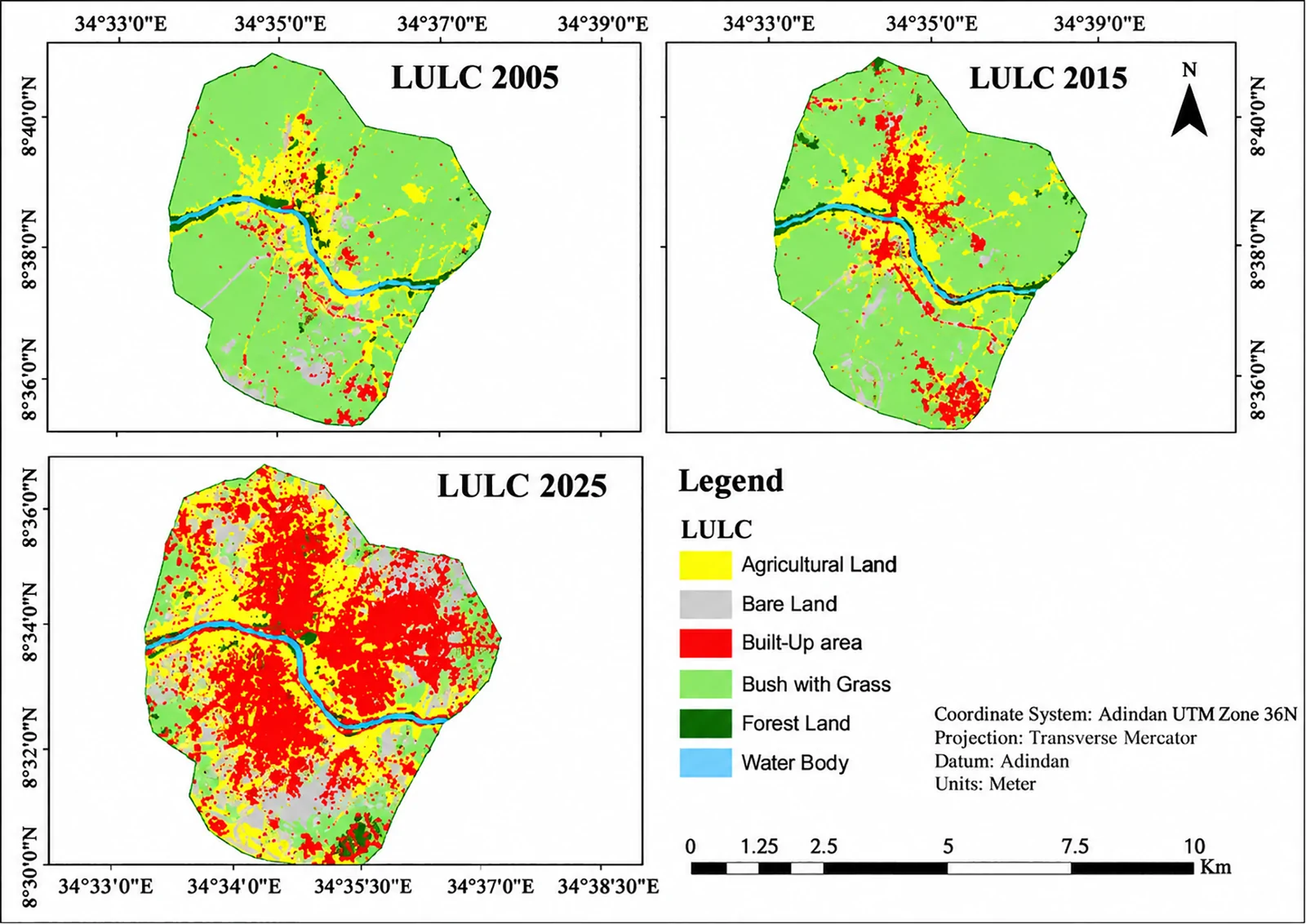
Impact of land-use/land-cover (LULC) change on urban expansion trends in Gambella Town. Figure prepared by the authors based on the LULC change analysis conducted in this study. This is an original author-generated figure and was not reproduced or adapted from a previously published figure.

As the table below from the 2015 classified image, here also, the percentage coverage of Bush with Grass (Bush land mixed with grassland) is higher it covers 64.4%. The LULC classification for 2015 from the TM satellite image (Table 7 and Fig 11) showed that Bush with Grass makes up 64.4% (3,053 ha.), Agricultural land covers 15.9% (754 Ha.), bare land covers 6.8% (323 ha.), Built-Up area covers 9.6% (456 ha.), Water Body (river and ponds) covers 1.6% (77 ha), and Forest Land holds 1.7% (81 ha), of the district in 2015. Bush with Grass and Agricultural land are the dominant cover types in the district, while water bodies, Forest land and Built-Up area accounted for a very small percentage in 2015

As the Table 7 and Fig 11 shows for the 2025 classified image, the percentage coverage of agricultural land makes up it covers 36.9% (1,750 ha). The LULC classification for 2025 from the TM satellite image (Fig 11) in the 2025 the classification showed that Built-Up area is makeup it covers 41.3% (1,956 ha), bare land covers 4.9% (234 ha.), Agricultural Land covers 36.9% (1,750 ha.), Water Body (river and ponds) covers 1.7% (82 ha), and Forest Land holds 1.5% (70 ha), of the district in 2025. Built-Up area and Agricultural land are the dominant cover types in the district due to the natural population increases, migration from rural to urban and fertility of the soil for cultivation the crops and cotton, while water bodies and Forest land accounted for a very small percentage in 2025 (Fig 11).

### Accuracy assessment

#### Accuracy assessment for LULC mapping of 2005.

The Confusion Matrix showed that both commission and omission error and also the majority of the pixels were misclassified to one another and this is due to spectral similarities for the vegetation. The overall accuracy and kappa coefficient of this period of classification results 90% and 82 and accuracy of each LULC classes (Table 8).

**Table 8:** Description of the accuracy assessment for LULC mapping of 2000.

Class Name	Water Body	Forest Land	Agricultural Land	Bush with Grass	Built-Up area	Bare Land	Total	U_Accuracy	Kappa
**Water Body**	4.00	0.00	0.00	0.00	0.00	0.00	4.00	1.00	0.00
**Forest Land**	0.00	4.00	1.00	0.00	0.00	0.00	5.00	0.80	0.00
**Agricultural Land**	1.00	2.00	27.00	0.00	1.00	1.00	32.00	0.84	0.00
**Bush with Grass**	3.00	0.00	6.00	153.00	3.00	0.00	165.00	0.93	0.00
**Built-Up area**	0.00	0.00	0.00	0.00	8.00	2.00	10.00	0.80	0.00
**Bare Land**	1.00	0.00	1.00	2.00	1.00	29.00	34.00	0.85	0.00
**Total**	9.00	6.00	35.00	155.00	13.00	32.00	250.00	0.00	0.00
**P_Accuracy**	0.44	0.67	0.77	0.99	0.62	0.91	0.00	0.90	0.00
**Kappa**	0.00	0.00	0.00	0.00	0.00	0.00	0.00	0.00	0.82

#### Accuracy assessment for LULC mapping of 2015.

Accuracy of LULC classification in 2015, Table 9 displays the accuracy of each LULC class, and the total accuracy and kappa coefficient for this period of classification results are 90% and 81, respectively.

**Table 9:** Description of the accuracy assessment for LULC mapping of 2010.

Class Name	Water Body	Forest Land	Agricultural Land	Bush with Grass	Built-Up area	Bare Land	Total	U_Accuracy	Kappa
**Water Body**	9.00	0.00	0.00	1.00	0.00	0.00	**10.00**	**0.90**	**0.00**
**Forest Land**	0.00	8.00	1.00	1.00	0.00	0.00	**10.00**	**0.80**	**0.00**
**Agricultural Land**	0.00	1.00	29.00	1.00	1.00	1.00	**33.00**	**0.88**	**0.00**
**Bush with Grass**	2.00	4.00	3.00	172.00	1.00	2.00	**184.00**	**0.93**	**0.00**
**Built-Up area**	0.00	1.00	0.00	1.00	13.00	0.00	**15.00**	**0.87**	**0.00**
**Bare Land**	1.00	1.00	0.00	0.00	2.00	7.00	**11.00**	**0.64**	**0.00**
**Total**	**12.00**	**15.00**	**33.00**	**176.00**	**17.00**	**10.00**	**263.00**	**0.00**	**0.00**
**P_Accuracy**	**0.75**	**0.53**	**0.88**	**0.98**	**0.76**	**0.70**	**0.00**	**0.90**	**0.00**
**Kappa**	**0.00**	**0.00**	**0.00**	**0.00**	**0.00**	**0.00**	**0.00**	**0.00**	**0.81**

#### Accuracy assessment for LULC mapping of 2025.

The Confusion Matrix showed that both commission and omission error and also the majority of the pixels were misclassified to one another and this is due to spectral similarities for the vegetation. User’s accuracy, producer’s accuracy, kappa coefficient and overall accuracy value for the classified image of 2025. The overall accuracy and kappa coefficient of this period of classification results 89% and 85 and accuracy of each LULC classes (Table 10).

**Table 10:** Description of the accuracy assessment for LULC mapping of 2025.

Class Name	Water Body	Forest Land	Agricultural Land	Bush with Grass	Built-Up area	Bare Land	Total	U_Accuracy	Kappa
**Water Body**	7.00	1.00	1.00	0.00	0.00	0.00	9.00	0.78	0.00
**Forest Land**	1.00	6.00	0.00	1.00	1.00	0.00	9.00	0.67	0.00
**Agricultural Land**	1.00	1.00	41.00	2.00	1.00	2.00	48.00	0.85	0.00
**Bush with Grass**	0.00	0.00	2.00	31.00	0.00	0.00	33.00	0.94	0.00
**Built-Up area**	1.00	2.00	0.00	2.00	77.00	2.00	86.00	0.90	0.00
**Bare Land**	2.00	1.00	0.00	1.00	1.00	60.00	65.00	0.92	0.00
**Total**	**12.00**	**11.00**	**44.00**	**37.00**	**80.00**	**64.00**	**250.00**	**0.00**	**0.00**
**P_Accuracy**	**0.58**	**0.55**	**0.93**	**0.84**	**0.96**	**0.94**	**0.00**	**0.89**	**0.00**
**Kappa**	**0.00**	**0.00**	**0.00**	**0.00**	**0.00**	**0.00**	**0.00**	**0.00**	**0.85**

### Land use/Land cover change three time periods

In the period between 2005 and 2015, Agricultural Land increased by 120 ha; (2.5%), Barren Land decreased by 142.98 ha; (−3.0%), Bush with grass decreased by 322 ha; (−6.7%), Built-Up area increased by 363 ha; (7.6%), Forest Land decreased by 12 ha, (−0.3), and Water bo + dy decreased by 6. ha, (−0.1%) respectively.

The result for the second period 2015 and 2025, Agricultural Land increased by 996 ha; (21.0%), Barren Land decreased by 89 ha; (−1.9%), Bush with grass decreased by 2,401 ha; (50.7%), Built-Up area increased by 1,500 ha; (31.7%), Forest Land decreased by 11 ha; (−0.2), and Water body increased by 5.ha (0.1%) respectively.

The rate of change between three decades which is between 2005 and 2025 indicated that Agricultural land, and Built-Up area is increased by 1,750 ha; (36.9%) 1,956 ha; which is (41.3%) respectively. The remaining, bare land, Bush with grass, water body and Forest land decreased by 234 ha; (4.9%) 652 ha; (13.7%) 6 ha (0.01%) and 70 ha; (1.5%) respectively, (Table 11).

**Table 11:** Description of the land cover change rate over three time periods.

No	Class Name	2000-2010		2010-2023		2000-2013	
		Rate ha.	%	Rate ha.	%	Rate ha.	%
1	Water Body	−6.56	−0.1	5	0.1	−1	−0.02
2	Forest Land	−12	−0.3	−11	−0.2	−23	−0.5
3	Agricultural Land	120	2.5	996	21.0	1,750	36.9
4	Bush with Grass	−322	−6.7	−2,401	−50.7	−652	−13.7
5	Built-Up area	363	7.6	1,500	31.7	1,956	41.3
6	Bare Land	−142.98	−3.0	−89	−1.9	−234	−4.9

+ Increase in the rate of land cover class – Decrease in the rate of land cover class.

### Land use/Land covers change in Gambella Town from 2005 to 2025

The LULC transition matrix (Table 12) shows the conversions among land-cover classes between 2005–2025. In particular, the Built-Up area increased mainly through conversion of Bush with Grass (1,137.83 ha), Agricultural Land (223.04 ha), and Bare Land (189.51 ha), while smaller areas were converted from Forest Land (28.01 ha) and Water Body (22.89 ha). This demonstrates the major land-cover sources contributing to urban expansion in Gambella Town. Major LULC change matrix below showed that the change study covered the 30-year period from 2005 to 2025. LULC change analysis using TM Landsat imagery revealed that, between 2005 and 2025, according to Table 12, of the predominant Bush with grass, 568.49 Ha. Remained, and a significant portion of it, about 1,137.83, was converted to Built-Up area and other cover types in varying amounts. Conversely, a significant portion of Bare land about 189 Ha; was transformed to Built-Up area of it, with the remaining portion being used for various kinds of cover. From 2005 to 2025, the amount of Agricultural Land that remained was around 278 Ha; the remainder was divided into other cover types, with 223 Ha; And 35.50 Ha; of the Agricultural Land being converted to Built-Up area and Bare Land, respectively. The percentage of Forest Land that changed from 2005 to 2025 was likewise substantial, with 28.01 Ha, going to Built-Up area, 32.84 Ha. To Agricultural land and the remaining percentage going to other land cover types.

**Table 12:** Description of the LULC class conversions in percent between 2005 and 2025.

Class Name		2025						
		Agricultural Land	Bare Land	Built-Up area	Bush with Grass	Forest Land	Water Body	Total
2005	**Agricultural Land**	**278**	35.50	223.04	47.29	38.21	20.81	642.86
	**Bare Land**	113.40	**78.00**	189.51	57.53	15.86	9.73	464.03
	**Built-Up area**	21.80	9.86	**41.74**	11.81	4.32	3.18	92.72
	**Bush with Grass**	728.39	903.03	1137.83	**568.49**	30.61	0.39	3368.74
	**Forest Land**	32.84	4.62	28.01	4.63	**6.29**	16.83	93.23
	**Water Body**	20.43	0.69	22.89	1.06	6.61	**30.81**	82.48
	**Total**	1194.86	1031.70	1643.03	690.82	101.89	81.77	**4,744**

### LULC class conversions 2005 and 2025

As you see in the Fig 12 it indicates that the urban sprawl changes in the Gambella Town with in the two decade. The conversion map of 2005–2025 clarify the changes between the Agricultural land with Bare land, Build up area, Bush with grass, forest land, water body and others Land use and covers classes.

**Fig 12:**
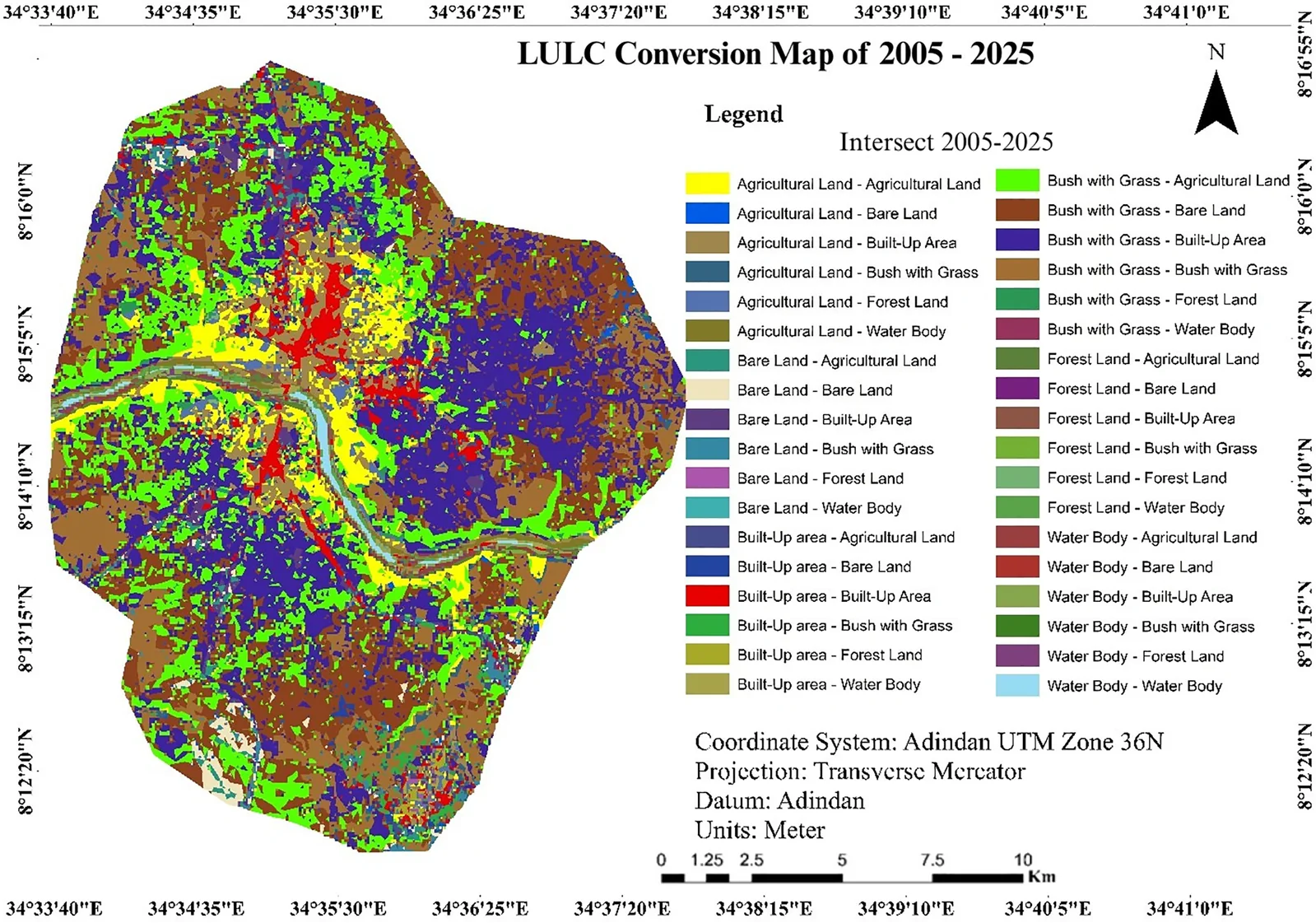
LULC conversion in Gambella Town between 2005 and 2025. Figure prepared by the authors based on the LULC classification and change-detection analysis conducted in this study. This is an original author-generated figure and was not reproduced or adapted from a previously published figure.

### Impact on the urban expansion trend of LULCC in Gambella town (2005–2025)

The study indicate that this two decade trend of LULCC on urban expansion in the Gambella town have both positives and negatives impacts on the community who are living in the study area. The positive impact on the urban expansion majority of peoples they can enjoy of having many position of services like good health, education, batter job opportunity and good life of social standard and the other hand it effects the Agro ecology society because their Agricultural land is converted to the residents area or build up this may lead them not adapt their living standard because they depend on the agriculture as the main sources of their economy income.

Urban expansion has also the negatives impact on the environment to loss the bio diversity, soil erosion, land degradation, air pollution and wildlife, the endemics wild animal are migrating to the neighbor regions and country as the region share the borders with South Sudan in the west due to the unplanning of urban expansion.

### Urban expansion map of Gambella Town from 2005 and 2025

The Fig 13 below on environmental degradation, urban expansion can have an adverse effect on ecosystems and biodiversity by destroying habitat, increasing pollution, and reducing green areas. However the total land area, only 968.87 hectares were 11% inhabited in 2005. In 2015, this was expanded to 2786.64 acres of land. The increase was 33%. A significant increase of 33% from 2015 and 56% from 2025 was noted in 2025, with a total land cover of 4744 hectare. A significant rate of built-up land expansion was seen between 2005 and 2025, a 20-year difference and a 39.3% increase, in addition to the time separation between the study periods due to the fast growth of the town many indigenous are seeking for the settlements land to differences site location (Fig 13)

**Fig 13:**
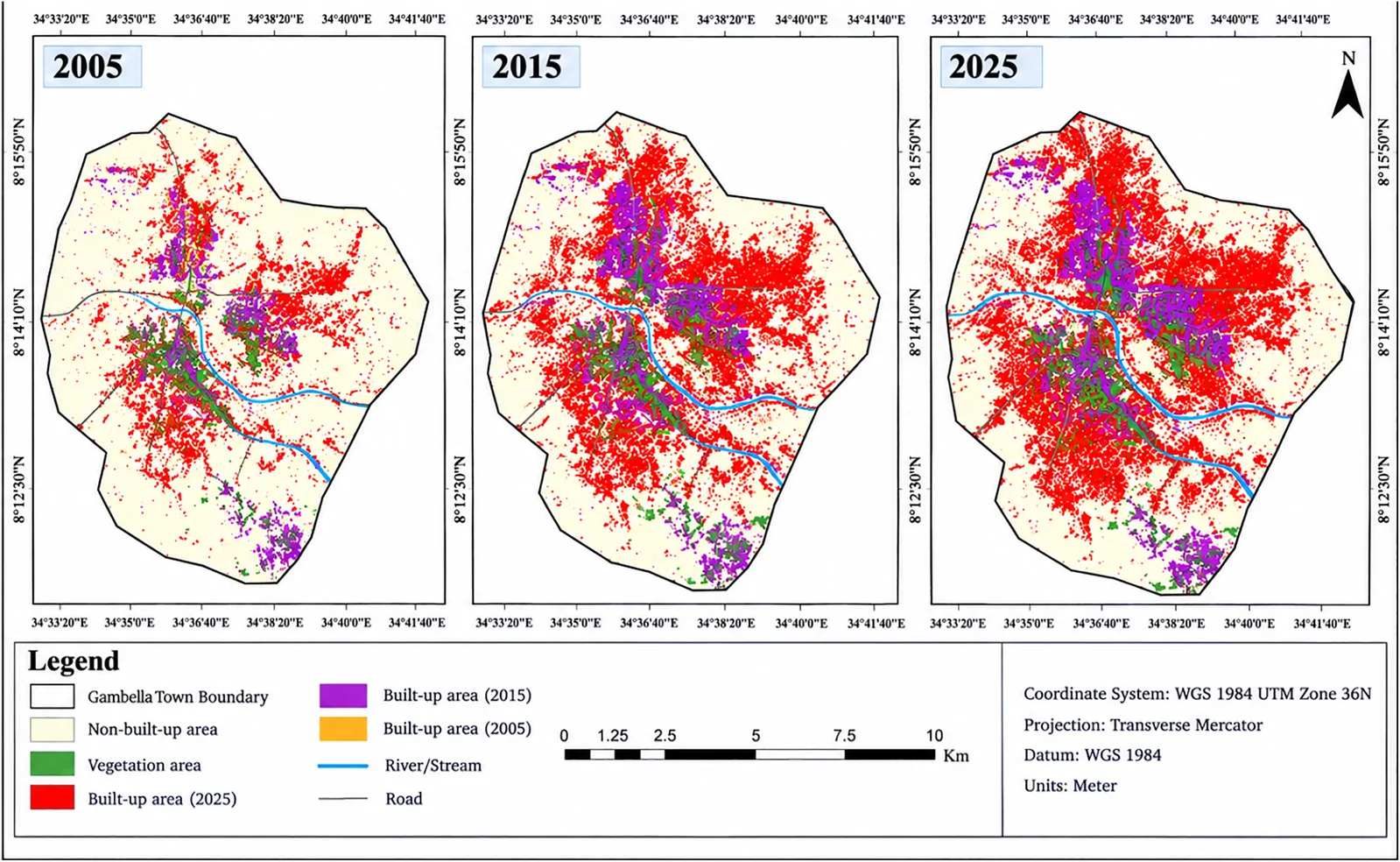
Urban expansion in Gambella Town. Figure prepared by the authors based on the LULC classification and spatial analysis conducted in this study. This is an original author-generated figure and was not reproduced or adapted from a previously published figure.

#### Net changes of land use land cover from 2005–2025 in Gambella town.

The data presented illustrate changes in land use over time across several categories. In 2005, the area of water bodies was 83 hectares, which decreased slightly to 77 hectares by 2015, then increased to 82 hectares in 2025, resulting in a net change of −1 hectare. Forest land declined from 93 hectares in 2005–81 hectares in 2015 and further to 70 hectares by 2025, reflecting a total decrease of 23 hectares. In contrast, agricultural land experienced significant growth, expanding from 634 hectares in 2005–754 hectares in 2015 and surging to 1,750 hectares by 2025, resulting in a net increase of 1,116 hectares. The area of bush with grass saw a dramatic decline, dropping from 3,375 hectares in 2005–3,053 hectares in 2015 and plummeting to 652 hectares by 2025, which amounts to a loss of 2,723 hectares. Built-up areas expanded rapidly, growing from 93 hectares in 2005–456 hectares in 2015 and further increasing to 1,956 hectares by 2025, marking a net change of 1,866 hectares. Lastly, bare land decreased from 466 hectares in 2005–323 hectares in 2015 and further to 234 hectares in 2025, showing a net decrease of 232 hectares. The Fig 14 shows the decrease and increase of the land use classification net changes from the years of 2005–2025 in Gambella town.

**Fig 14:**
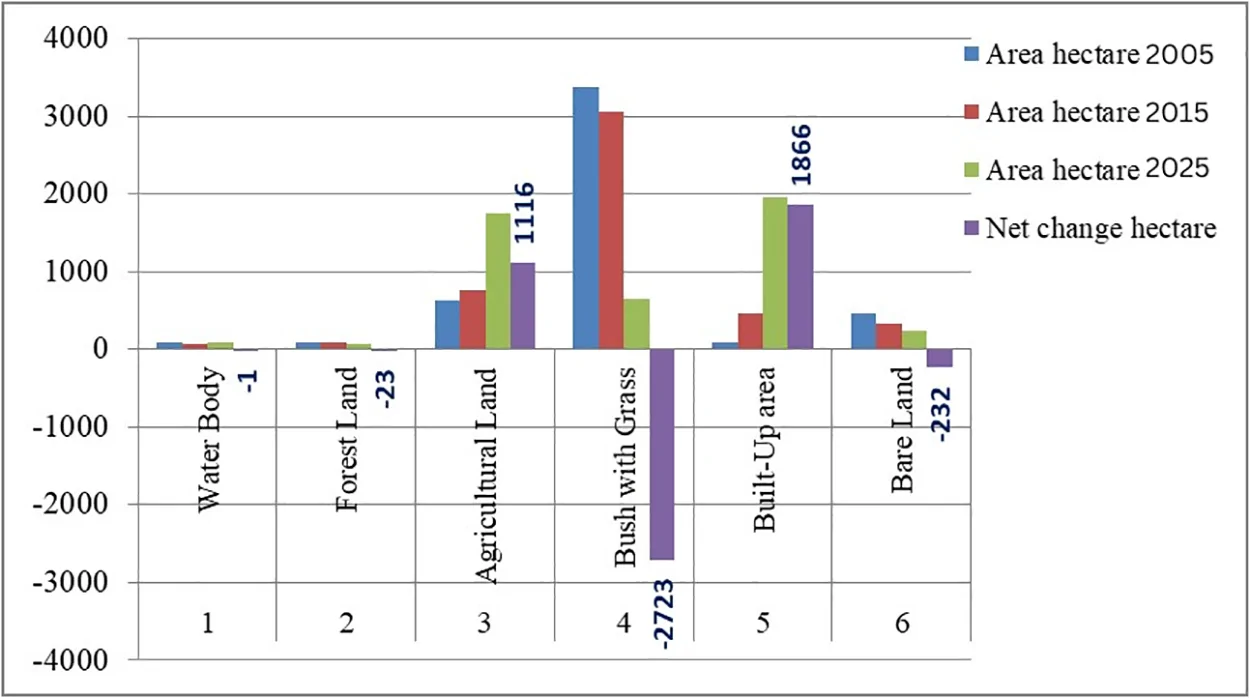
Net changes of land use land cover from 2005-2025 in Gambella town.

### Receiver Operating Characteristic (ROC) curve analysis

The machine learning model achieved an Area Under the Curve (AUC) value of 0.852 (85.2%) based on the Receiver Operating Characteristic (ROC) analysis, with 200 validation points used for evaluation (Fig 15). This result indicates a high discriminatory capability of the model in effectively distinguishing urban and non-urban areas in Gambella Town. AUC values between 0.80 and 0.90 suggest fair classification accuracy, whereas values above 0.90 show exceptional performance, according to well recognized performance metrics [37]. Typical of machine learning classifiers using finite validation datasets, the ROC curve has a distinctive step-like structure. There are discrete increases in the true positive rate (TPR) and corresponding changes in the false positive rate (FPR) at each step that corresponds to changes in the decision threshold, where additional pixels are classed as urban.

**Fig 15:**
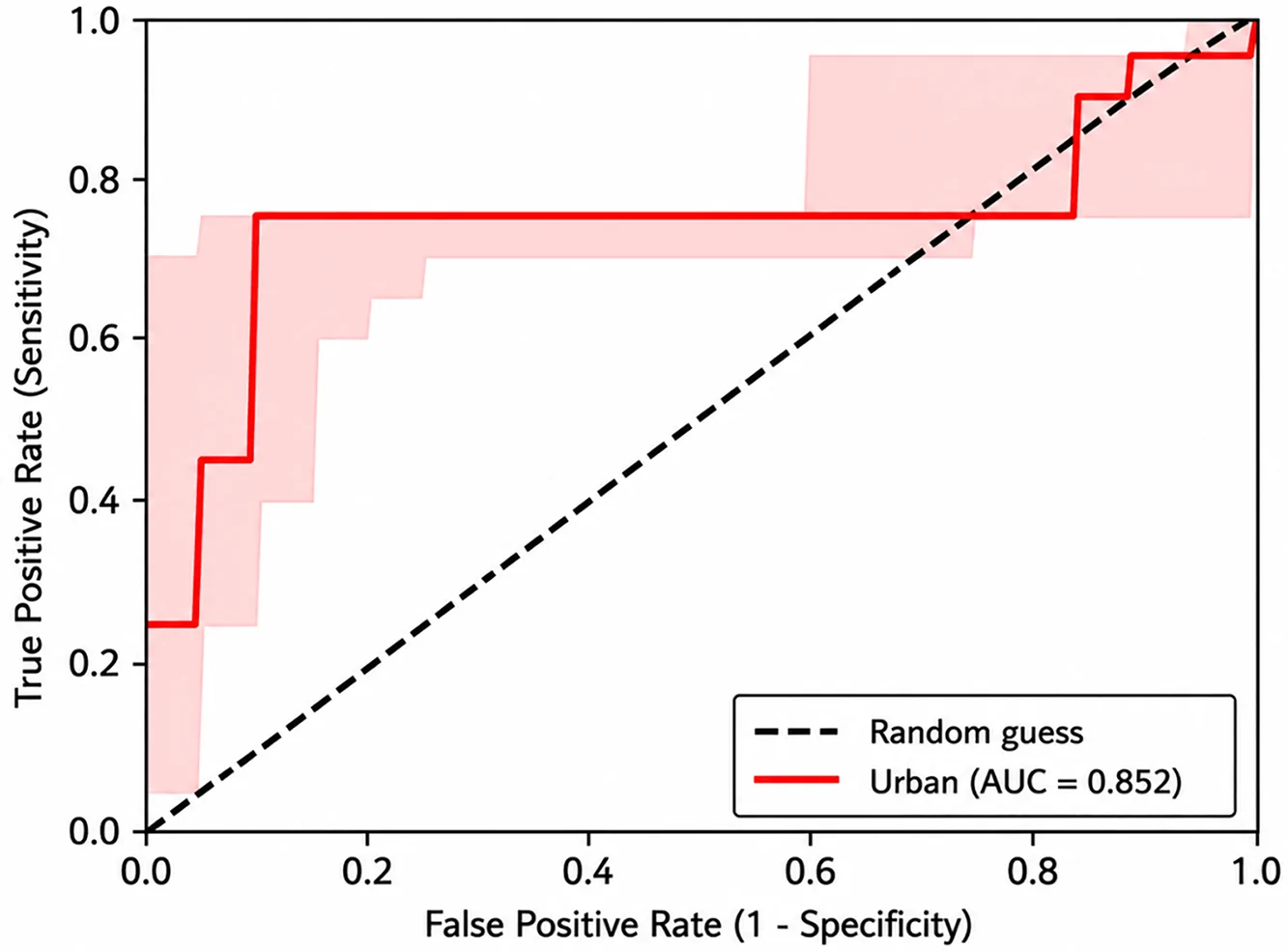
Map of the Receiver Operating Characteristic (ROC) curve analysis.

Strong sensitivity during the early stages of classification is demonstrated by the sharp spike seen at low false positive rates, which shows that the model accurately recognizes a significant percentage of urban pixels while introducing comparatively few false alarms. Because of the spectral overlap between urban surfaces, bare land, and sparsely vegetated areas that frequently occur in the diverse peri-urban landscapes of Gambella, the extended plateau in the middle portion of the curve indicates that, over a range of thresholds, further increases in sensitivity require accepting additional false positives. The ROC curve constantly stays above the diagonal random-guess line in spite of these difficulties, demonstrating that the classifier outperforms chance at every decision threshold. Mixed pixels, seasonal land-cover variability, and the spectral similarity of some land-cover classes in medium-resolution Landsat imagery are the main causes of the remaining discrepancy between the observed AUC (0.852) and the ideal value of 1.0, which denotes a moderate degree of classification uncertainty. Previous ROC features and AUC values for machine learning-based urban mapping have been reported in previous studies. For instance, Chen et al. [38–41] obtained AUC values between 0.84 and 0.89 for urban expansion prediction using geospatial machine learning techniques, whereas Arabameri et al. [42–45] and Pham et al. [46] reported AUC values exceeding 0.85 for urban growth susceptibility modeling using Random Forest-based approaches.

Similarly, Liu et al. [47–50] showed that in quickly developing countries, AUC values of approximately 0.85 offer dependable discrimination for urban growth modeling. Consequently, the obtained AUC of 85.2% supports the application of machine learning and geospatial technologies in urban planning, land-use management, and sustainable development by providing a strong and dependable framework for capturing the spatiotemporal dynamics of urban expansion in Gambella Town.

## Discussions

Gambella Town’s land-use and land-cover (LULC) changes are closely related to several socioeconomic variables, chief among them the migration of rural to urban residents looking for safety and opportunity in the host town. Due to the increased demand for land for residents and habitation purposes brought about by this demographic transition, there have been major environmental effects. Technology, environmental changes, economic development, and human population expansion are all identified by Getu, K., & Bhat, H. G. [2,11] as important factors that influence LULC variability. These elements are reshaping the environment and the socioeconomic structure of Gambella as they appear.

The rapid expansion of built-up areas observed in Gambella Town is consistent with urban land-cover changes documented in other Ethiopian cities. Studies in Addis Ababa, Bahir Dar, and Hawassa have documented substantial urban expansion accompanied by changes in agricultural and vegetated land [51–55]. Similar trends have been reported in rapidly growing African cities such as Nairobi, Dar es Salaam, and Accra, where urban expansion has increasingly transformed surrounding agricultural and natural landscapes [6,56,57]. These similarities indicate that the transformation observed in Gambella reflects a broader pattern of urbanization across Africa, although the simultaneous expansion of agricultural land in Gambella highlights the influence of local land availability, environmental conditions, migration, and agricultural investment on its LULC dynamics.

The settlement area increased dramatically from 93 hectares (2.0%) in 2005–1,956 hectares (41.3%) in 2025, highlighting the pressing need for infrastructure and housing to support the expanding population. This urban sprawl is indicative of a larger pattern seen in areas that are quickly urbanizing, where the growth of human settlements frequently comes at the price of natural ecosystems. The pressure on land resources can lead to habitat loss, increased pollution, and challenges in water management, as noted by studies on urbanization impacts [11,15,58,59].

The remarkable increase in agricultural land from 634 hectare (13.4%) in 2005–1,750 hectares (36.9%) in 2025 highlights, Gambella’s appeal as a fertile agricultural zone this expansion can be attributed to the area’s rich soil and favorable climate, attracting both local and foreign investors seeking to capitalize on agricultural opportunities. The shift towards commercial agriculture, particularly for crops like beans, grains, cotton, and sugarcane, is reflective of a global trend where fertile lands are increasingly utilized for large-scale agricultural production.

The decline of bush with grass from 3,375 hectares (71.1%) in 2005–652 hectares (13.7%) by 2025 signals a significant loss of biodiversity and natural habitat. This loss poses risks not only to local wildlife but also to ecosystem services that communities depend on, such as soil stability and water regulation. As noted [10,11] the conversion of natural landscapes bush with grass and others land use classes to urban use can lead to ecological imbalances, exacerbating environmental degradation and undermining resilience to climate change.

Additionally, the reduction in bare land from 466 hectares (9.8%) in 2005–234 hectares (4.9%) in 2025 reflects a broader trend of land reclamation for productive uses. While this may indicate positive land utilization, it also suggests a shift away from less productive or unmanaged land, further emphasizing the need for careful land-use planning and management. The fluctuations in forest cover, decreasing from 93 hectares (2.0%) in 2005–70 hectares (1.5%) in 2025, reveal the precarious balance between urban expansion, agriculture, and conservation efforts.

The stable area of water bodies, from 83 hectares (1.7%) in 2005–82 hectares (1.7%) in 2025, raises important questions about water resource management amidst growing demands. Population growth inevitably leads to increased water consumption, necessitating sustainable water management practices to ensure that both human and ecological needs are met. The consistent presence of water bodies highlights their critical role in the local ecosystem, but also the potential for conflicts over water access as urban and agricultural demands increase.

Overall, socio demographic data and the classified satellite images provide compelling evidence of LULC changes over the past two decades in Gambella Town. These transformations reflect complex interactions between human activity and environmental systems have both positives and negatives impacts on socio economic and environment, underscoring the need for integrated planning that considers ecological sustainability alongside socio-economic development.

## Conclusions and recommendations

This study assessed land use/land cover (LULC) dynamics and the socioeconomic and environmental implications of urban expansion in Gambella Town between 2005 and 2025 using satellite imagery, GIS-based analysis, and socioeconomic data. The results demonstrate substantial landscape transformation during the study period, particularly a pronounced expansion of built-up areas, which increased from 93 ha (2.0%) in 2005–1,956 ha (41.3%) in 2025. Agricultural land also increased from 634 ha to 1,750 ha, while bush with grass declined substantially from 3,375 ha to 652 ha. Bare land and forest land also decreased, indicating continued transformation of natural and semi-natural land covers.

The findings indicate that the rapid expansion of built-up areas has altered the spatial structure of Gambella Town and increased pressure on agricultural and natural land resources. The conversion of bush with grass and other natural land covers may contribute to habitat loss and environmental degradation, while changes in agricultural land may affect local livelihoods and food-security conditions. The study therefore demonstrates the importance of managing urban growth in a manner that considers both development needs and the conservation of productive and ecologically important land.

To promote sustainable urban development, urban authorities should strengthen land-use zoning and development control, particularly around agricultural and environmentally sensitive areas. Future urban planning should be supported by regularly updated satellite imagery, GIS-based monitoring, and evidence-based land allocation. Integrating environmental protection, livelihood considerations, and infrastructure planning into future urban development strategies will help guide Gambella Town toward more sustainable and spatially balanced growth.
